# lncRNA Oip5-as1 inhibits excessive mitochondrial fission in myocardial ischemia/reperfusion injury by modulating DRP1 phosphorylation

**DOI:** 10.1186/s11658-024-00588-4

**Published:** 2024-05-14

**Authors:** Xiaowei Niu, Jingjing Zhang, Shuwen Hu, Wenhui Dang, Kaiwen Wang, Ming Bai

**Affiliations:** 1 Department of Cardiology, The First Hospital of Lanzhou University, Lanzhou, Gansu 730000 China; 2 Gansu Key Laboratory of Cardiovascular Diseases, The First Hospital of Lanzhou University, Lanzhou, Gansu 730000 China; 3 Gansu Clinical Medical Research Center for Cardiovascular Diseases, The First Hospital of Lanzhou University, Lanzhou, Gansu 730000 China; 4 The First School of Clinical Medicine, Lanzhou University, Lanzhou, Gansu 730000 China; 5 Medical Genetics Center, Gansu Provincial Central Hospital/Gansu Provincial Maternity and Child-Care Hospital, Lanzhou, Gansu 730000 China; 6 Gansu Provincial Clinical Research Center for Birth Defects and Rare Diseases, Lanzhou, Gansu 730000 China

**Keywords:** Long non-coding RNAs, Oip5-as1, Myocardial ischemia/reperfusion injury, Mitochondrial fission, Calcineurin, AKAP1, DRP1

## Abstract

**Background:**

Aberrant mitochondrial fission, a critical pathological event underlying myocardial ischemia/reperfusion (MI/R) injury, has emerged as a potential therapeutic target. The long non-coding RNA (lncRNA) Oip5-as1 is increasingly recognized for its regulatory roles, particularly in MI/R injury. However, its precise mechanistic role in modulating mitochondrial dynamics remains elusive. This study aims to elucidate the mechanistic role of Oip5-as1 in regulating mitochondrial fission and evaluate its therapeutic potential against MI/R injury.

**Methods:**

To simulate in vitro MI/R injury, HL-1 cardiomyocytes were subjected to hypoxia/reoxygenation (H/R). Lentiviral vectors were employed to achieve overexpression or knockdown of Oip5-as1 in HL-1 cells by expressing Oip5-as1 or shRNA targeting Oip5-as1, respectively. The impact of Oip5-as1 on mitochondrial dynamics in HL-1 cells was assessed using CCK-8 assay, flow cytometry, immunofluorescence staining, and biochemical assays. MI/R injury was induced in mice by ligating the left anterior descending coronary artery. Conditional knockout mice for Oip5-as1 were generated using the CRISPR/Cas9 genome editing technology, while overexpression of Oip5-as1 in mice was achieved via intramyocardial administration of AAV9 vectors. In mice, the role of Oip5-as1 was evaluated through echocardiographic assessment, histopathological staining, and transmission electron microscopy. Furthermore, Western blotting, RNA pull-down, RNA immunoprecipitation, and co-immunoprecipitation assays were conducted to investigate Oip5-as1’s underlying mechanisms.

**Results:**

The expression levels of Oip5-as1 are significantly decreased in MI/R-injured HL-1 cells and myocardium. In HL-1 cells undergoing H/R injury, overexpression of Oip5-as1 attenuated excessive mitochondrial fission, preserved mitochondrial functionality, and reduced cellular apoptosis, while knockdown of Oip5-as1 exhibited the opposite effects. Furthermore, in a mouse model of MI/R injury, overexpression of Oip5-as1 diminished mitochondrial fission, myocardial infarct size and improved cardiac function. However, knockout of Oip5-as1 exacerbated myocardial injury and cardiac dysfunction, which were significantly reversed by treatment with a mitochondrial division inhibitor-1 (Mdivi-1). Mechanistically, Oip5-as1 selectively interacts with AKAP1 and CaN proteins, inhibiting CaN activation and subsequent DRP1 dephosphorylation at Ser637, thereby constraining DRP1’s translocation to the mitochondria and its involvement in mitochondrial fission.

**Conclusions:**

Our study underscores the pivotal role of Oip5-as1 in mitigating excessive mitochondrial fission during MI/R injury. The findings not only enhance our comprehension of the molecular mechanisms underlying MI/R injury but also identify Oip5-as1 as a potential therapeutic target for ameliorating MI/R injury.

**Graphical Abstract:**

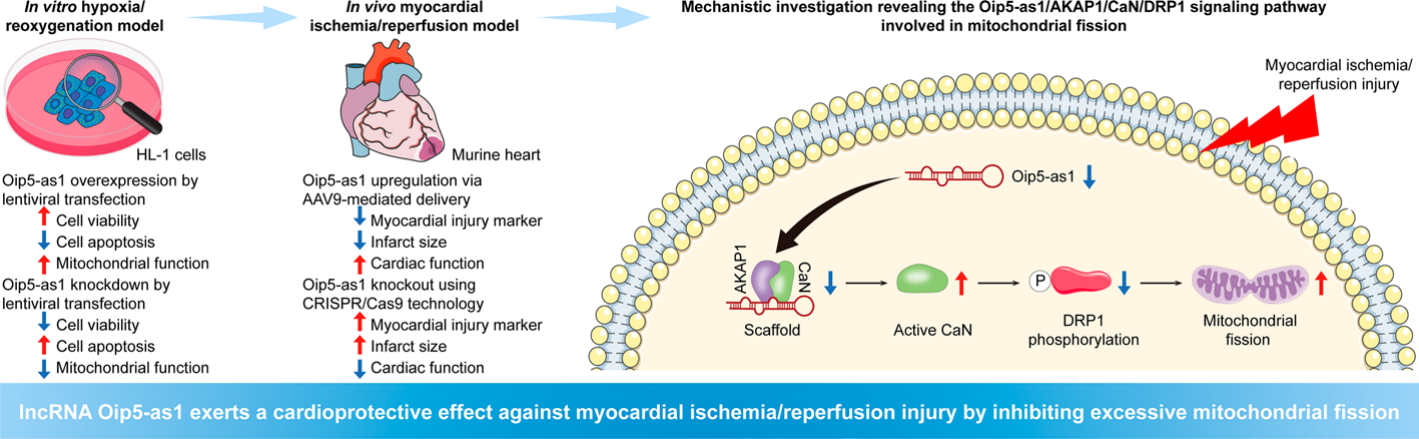

**Supplementary Information:**

The online version contains supplementary material available at 10.1186/s11658-024-00588-4.

## Background

Coronary artery disease remains the leading cause of morbidity and mortality worldwide [1]. Primary interventions, such as thrombolytic therapy and percutaneous coronary intervention, are critical in restoring blood flow in patients with this condition [2, 3]. Although these reperfusion treatments are essential for reducing cardiac injury by resupplying blood to ischemic myocardium, they paradoxically may also initiate myocardial ischemia/reperfusion (MI/R) injury [2, 3]. MI/R injury is known to induce cardiomyocyte death, arrhythmia, and cardiac dysfunction, leading to poor prognosis in patients [2, 3]. Previous studies have demonstrated that MI/R injury results from a complex interplay of pathological events upon reperfusion, including oxidative stress, inflammatory responses, and mitochondrial dysfunction [4]. Despite these insights, developing effective therapeutic agents to target MI/R injury remains challenging [2–5]. This underscores the need for a deeper understanding of its molecular mechanisms and the identification of novel therapeutic targets.

Mitochondria are dynamic organelles that regulate many important cellular processes, including metabolism, ATP generation, immune response and activation of cell death pathways [6]. The morphology of mitochondria changes dynamically in response to environmental cues, which in turn affects mitochondrial function [7, 8]. Mitochondrial dynamics, including the processes of fission and fusion, play critical roles in both physiological and pathological conditions [4, 6, 7]. In the healthy heart, a delicate balance between mitochondrial fission (division) and fusion (joining) is essential for maintaining optimal cellular function [7]. Mitochondrial fission facilitates the segregation and removal of damaged mitochondria, whereas mitochondrial fusion helps maintain mitochondrial function and genetic integrity [7]. In contrast, in the context of cardiac pathologies, especially MI/R injury, this balance is often disrupted [4, 6]. Excessive mitochondrial fission is observed, leading to mitochondrial fragmentation, impaired function, and increased susceptibility to cell death [9–11]. This pathological shift towards mitochondrial fragmentation has been linked to the activation of key fission proteins, such as dynamin-related protein 1 (DRP1), and is associated with increased oxidative stress and apoptosis in cardiac cells [11]. Genetic ablation of DRP1 has been shown to restrict MI/R injury in mice [12, 13], a concept further validated by pharmacological inhibitors [10, 14]. Despite its therapeutic potential, the physiological roles of mitochondrial fission should be considered when developing treatments targeting DRP1 [6].

Previous studies have identified various post-translational modifications of DRP1, such as phosphorylation, SUMOylation, acetylation, O-GlcNAcylation, and S-nitrosylation, which regulate its activity and influence the balance between fission and fusion [15, 16]. However, the precise means of achieving sophisticated post-translational regulation of DRP1 in specific cellular contexts remains an area of active research. Understanding these mechanisms is not only pivotal for deciphering the complex interplay between mitochondrial dynamics and cellular health. It is also important for designing targeted treatments that selectively target pathological mitochondrial fission without compromising physiological division [15].

Long noncoding RNAs (lncRNAs), a class of multifunctional transcripts longer than 200 nucleotides with minimal protein-coding capacity, play a pivotal role in various biological processes, including RNA splicing, protein localization, and protein modification [17–19]. In the context of cardiac diseases, lncRNAs have been demonstrated to be key players. Several lncRNAs are implicated in regulating MI/R injury, such as lncRNA CIRKIL [20], which exacerbates cardiomyocyte apoptosis by influencing the nuclear translocation of Ku70, and lncRNA CPhar [21], protecting against apoptosis by modulating the interaction between DDX17 and C/EBPβ. Despite these insights, the specific role of lncRNAs in DRP1-mediated mitochondrial fission during MI/R injury remains less explored, presenting a significant opportunity for future research.

Our recent study has focused on the lncRNA Opa-interacting protein 5-antisense transcript 1 (Oip5-as1), which we identified as being downregulated in MI/R injury [22]. Unlike most lncRNAs, Oip5-as1 is characterized by high nucleotide sequence homology across various mammalian species, including humans [23–25]. Both transcriptomics and experimental data reveal a notable enrichment of Oip5-as1 in striated muscles, such as the diaphragm, skeletal muscle, and heart, as well as in the brain, highlighting its potential significance in cardiac pathologies [25]. A study suggests that reduced Oip5-as1 expression exacerbates heart failure in the context of cardiac pressure overload [25]. Furthermore, Oip5-as1 is implicated in the regulation of fibrosis, mitosis, and cell proliferation across various chronic diseases and cancers [26]. Predominantly, the functions of Oip5-as1 are linked to its interactions with microRNAs, acting as a competing endogenous RNA (ceRNA) [26]. The broad impact of Oip5-as1 in both physiological and disease states underlines its importance as a potential therapeutic target. Consequently, detailed exploration of the molecular mechanisms through which Oip5-as1 exerts its effects in the heart would offer promising avenues for the development of novel therapeutic strategies for MI/R injury.

In this study, we utilized gain- and loss-of-function experiments to demonstrate that Oip5-as1 overexpression attenuates excessive mitochondrial fission and preserves mitochondrial function during MI/R injury, while Oip5-as1 knockdown leads to the opposite effect. Mechanistically, we discovered that Oip5-as1 binds specifically to a kinase anchor protein 1 (AKAP1) and calcineurin (CaN) proteins, inhibiting the release of active CaN and its subsequent dephosphorylation of DRP1. As a result, this inhibition limits DRP1’s translocation to mitochondria and reduces its role in mitochondrial fission. Our findings provide a comprehensive understanding of the molecular interactions and pathways modulated by Oip5-as1, highlighting its potential as a therapeutic target for MI/R injury treatment.

## Materials and methods

### Cell culture and treatment

The murine HL-1 cardiomyocyte-like cell (Catalogue numbers: SCC065) was procured from Sigma-Aldrich (St. Louis, MO, USA) and cultured in Claycomb media (Sigma-Aldrich) enriched with 10% fetal bovine serum (Thermo Fisher Scientific, Waltham, MA, USA), 0.1 mM norepinephrine (Solarbio, Beijing, China), and 2 mM L-glutamine (Solarbio). The cells were incubated in a humidified atmosphere at 37 °C with 5% CO_2_.

To establish an in vitro model of MI/R injury, HL-1 cells were treated with glucose-free Hank’s balanced salt solution. The cells were then incubated in a HeraCell VIOS 160i incubator (Thermo Fisher Scientific) with a hypoxic gas mixture containing 1% O_2_, 5% CO_2_, and 94% N_2_ for 6 h. Following hypoxic treatment, the cells underwent reoxygenation for varied durations of 6, 12, and 24 h, respectively, in Claycomb medium under normoxic conditions (95% air and 5% CO_2_). In contrast, control cells were maintained in Claycomb medium in a humidified 5% CO_2_ incubator under normoxic conditions. In specific experiments, HL-1 cells were pre-treated with 50 μM mitochondrial division inhibitor-1 (Mdivi-1; Selleckchem, Houston, Texas, USA) or 1 μM calcineurin inhibitor FK506 (MedChemExpress, Monmouth Junction, NJ, USA) prior to hypoxia/reoxygenation (H/R) induction.

### Cell viability assay

Cell viability was determined using a Cell Counting Kit-8 (CCK-8; Biosharp, Hefei, Anhui, China) following the manufacturer’s protocol. In brief, HL-1 cells were distributed in 96-well plates at a concentration of 5000 cells for each well. Subsequent to the treatments, each well was supplemented with 10 μL of CCK-8 solution and the plates were subjected to a 37 °C incubation for a duration of 2 h. The optical density (OD) was measured at 450 nm using an Infinite M200 PRO microplate reader (Tecan, Männedorf, Switzerland).

### Assessment of cell apoptosis

Apoptosis analysis of HL-1 cells was performed using a FITC Annexin V Apoptosis Detection Kit (BD Biosciences, San Jose, CA, USA) by flow cytometry. HL-1 cells were digested and resuspended in 500 μL binding buffer. The resulting cell suspension was treated with 5 μL Annexin V-Fluorescein Isothiocyanate (Annexin V-FITC) and 5 μL of Propidium Iodide (PI) solutions for 15 min in the dark. The stained samples was subjected to flow cytometric analysis using a NovoCyte flow cytometer (ACEA Biosciences, San Diego, CA, USA). The percentage of apoptotic cells was determined by multiplying the ratio of Annexin V-positive cells to the total cell population by 100%.

### Lentiviral transfection and generation of stable cell lines

The overexpressed lentivirus for Oip5-as1 (oe-Oip5-as1), the short hairpin RNA (shRNA)-mediated lentivirus targeting Oip5-as1 (sh-Oip5-as1), and their respective negative control lentiviruses (oe-NC and sh-NC) were obtained from Genechem (Shanghai, China). HL-1 cells were transfected at a multiplicity of infection (MOI) of 100 for each virus, adhering to the manufacturer’s guidelines. The lentiviral-transduced cells were subsequently subjected to selection using 2 μg/mL puromycin (Genechem). The expression levels of Oip5-as1 in the puromycin-selected HL-1 cells were determined by real-time quantitative polymerase chain reaction (RT-qPCR). The details of shRNA sequences are listed in Additional file 1: Table S1.

### Small interfering RNA transfection

The small interfering RNAs (siRNAs) targeting AKAP1 (si-AKAP1) and the negative control siRNAs (si-NC) were designed and synthesized by GenePharma (Shanghai, China). A Lipofectamine 3000 reagent (Invitrogen, Waltham, MA, USA) was employed for transfection, following the manufacturer’s instructions. The efficacy of gene silencing was validated 48 h post-transfection using RT-qPCR and Western blot analysis. Target sequences for the AKAP1 gene are listed in Additional file 1: Table S1.

### Plasmid transfection

Genechem designed and synthesized the plasmid construct encoding the DRP1-S637A mutant, where DRP1 serine at position 637 was substituted with alanine. This mutation is known to abrogate the phosphorylation site of DRP1 at Ser637, leading to alterations in DRP1’s activity and mitochondrial fission processes [27]. Transfection was carried out using the Lipofectamine 3000 reagent, in line with the manufacturer’s guidelines. Subsequently, HL-1 cells were incubated for 48 h at 37 °C and 5% CO_2_, facilitating downstream analysis.

### Fluorescence in situ hybridization assay

Fluorescent Cy3-labeled probes targeting Oip5-as1, 18S RNA (as a positive control), and a scrambled RNA (as a negative control) were designed and synthesized by GenePharma. The fluorescence in situ hybridization (FISH) assay was conducted using a RNA FISH Kit supplied by GenePharma, following the manufacturer’s protocol. The images were acquired using a ZEISS LSM 880 confocal microscope (Oberkochen, Baden-Württemberg, Germany) with a 63 × magnification objective lens. The probe sequences are listed in Additional file 1: Table S1.

### Nuclear and cytoplasmic RNA fractionation

Nuclear and cytoplasmic RNA fractions were isolated from HL-1 cells using a Cytoplasmic and Nuclear RNA Purification Kit (Norgen Biotek, Thorold, ON, Canada) according to the manufacturer’s instructions. RT-qPCR was performed to assess the relative proportion of Oip5-as1 in the nuclear and cytoplasmic fractions. Actb expression was employed as a cytoplasmic control, whereas lncRNA Neat1 was utilized as a nuclear control. The primer sequences are available in Additional file 1: Table S1.

### Detection of mitochondrial reactive oxygen species

Mitochondrial reactive oxygen species (mitoROS) were assessed employing a MitoSOX Red Mitochondrial Superoxide Indicator (Thermo Fisher Scientific). HL-1 cells were cultured in six-well plates and incubated with the MitoSOX reagent at the optimal concentration of 5 μM for 10 min at 37 °C. Subsequent to staining, the cells were washed three times and then visualized using an Olympus IX71 microscope (Shinjuku, Tokyo, Japan). Images were acquired at 20 × magnification, allowing quantification of fluorescence intensity using ImageJ software (version 1.51 k; National Institutes of Health, Bethesda, MD, USA).

### Measurement of mitochondrial membrane potential

A Mitochondrial Membrane Potential Assay Kit with JC-1 (Beyotime, Shanghai, China) was used to monitor mitochondrial membrane potential (MMP). HL-1 cells were seeded in six-well plates and treated with 1 mL of JC-1 dye solution for 20 min at 37 °C. The stained cells were visualized by the Olympus microscope. Image analysis was conducted using the ImageJ software, and MMP quantification was determined by calculating the ratio of red to green fluorescence, corresponding to JC-1 aggregates and monomers, respectively.

### Mitochondrial morphology assessment

To observe the mitochondrial morphology, HL-1 cells were stained with a mitochondria-specific Tom-20 (translocase of outer mitochondria 20) antibody, following a method that has been widely described previously [28, 29] and used in our laboratory [30]. Briefly, HL-1 cells were fixed with 4% paraformaldehyde (PFA), permeabilized with Triton X-100, and blocked with a QuickBlock buffer (Beyotime). The primary antibody against Tom-20 (Cell Signaling Technology, Danvers, MA, USA) was applied overnight at 4 °C, followed by incubation with a CoraLite594 conjugated secondary antibody (Proteintech Group, Rosemont, IL, USA). Nuclei were stained with a DAPI solution (Beyotime) for 5 min.The stained cells were imaged with a 63 × oil objective on the ZEISS confocal microscopy. Image analysis was performed using Mito-morphology macro in the ImageJ software, based on the methods described by Fonseca et al. [28]. Mitochondrial size was assessed by the average area and perimeter, while shape was gauged by circularity. Fragmented and spherical mitochondria, indicative of fission, were identified by decreased size and increased circularity [28, 29].

### Immunofluorescence detection of DRP1 mitochondrial translocation

The translocation of DRP1 to mitochondria was assessed via immunofluorescence staining. HL-1 cells were cultured in laser confocal dishes and subjected to the specified experimental conditions. The cells were subsequently fixed with 4% PFA for a period of 15 min and permeabilized using Triton X-100 for 10 min. Following the blocking procedure with the QuickBlock buffer, the cells were incubated with a primary antibody against DRP1 (Cell Signaling Technology) at 4 °C overnight. After three washes with phosphate-buffered saline (PBS), the cells were further incubated for one hour in the dark with a CoraLite488-conjugated secondary antibody (Proteintech Group). Mitochondria staining was performed using a MitoTracker Red CMXRos kit (Thermo Fisher Scientific) per the manufacturer’s instructions. The cells were subsequently washed and mounted with the medium containing the DAPI to visualize nuclei. Imaging was conducted using the ZEISS confocal microscopy at a magnification of 63 × . The co-localization of DRP1 and mitochondria was analyzed using the ImageJ software.

### Isolation of mitochondrial and cytoplasmic fractions

Mitochondrial and cytoplasmic fractions were isolated with a Mitochondrial Extraction Kit (Solarbio) according to the manufacturer’s protocol. HL-1 cells or myocardial tissues were lysed in the ice-cold Lysis Buffer containing a Halt protease inhibitor cocktail (Thermo Scientific). Differential centrifugation at 1000 *g* for 5 min at 4 °C was performed twice to remove nuclei and intact cells from the homogenate. The resulting supernatant was further centrifugation at 12,000 *g* for 10 min at 4 °C to sediment the mitochondria, leaving the cytoplasmic fraction in the supernatant. To purify the mitochondrial fraction, it was washed with 0.5 mL Wash Buffer and centrifuged at 12,000 *g* for 10 min at 4 °C. This step was repeated until the mitochondrial pellet was free of cytoplasmic contamination. The integrity and purity of the isolated fractions were confirmed by Western blot analysis using COX IV (Cytochrome c oxidase subunit IV; Cell Signaling Technology) protein as a mitochondrial marker and Tubulin protein (Cell Signaling Technology) as a cytoplasmic marker.

### Determination of CaN activity

The activity of CaN was evaluated using a Calcineurin Assay Kit (Jiancheng, Nanjing, Jiangsu, China), in accordance with the manufacturer’s instructions. The absorbance was determined at a wavelength of 636 nm utilizing a JK 752N spectrophotometer (Jingke, Shanghai, China).

### Measurement of protein kinase A activity

The activity of protein kinase A (PKA) was assessed utilizing a PKA Colorimetric Activity Kit (Invitrogen), adhering to the manufacturer’s guidelines. Following the assay procedure, the colorimetric measurements were taken at a wavelength of 450 nm using the microplate reader.

### RNA pull-down assay

To identify Oip5-as1-associated protein partners, RNA pull-down assays were conducted. Oip5-as1 was synthesized in vitro using T7 RNA polymerase (Roche, Basel, Switzerland) and biotinylated with Biotin RNA Labeling Mix (Roche). The biotinylated Oip5-as1 was incubated with Dynabeads MyOne Streptavidin C1 beads (Invitrogen). Concurrently, the cell lysate was prepared using a RIPA buffer (Beyotime) enhanced with a RNase Inhibitor (Beyotime) and the Halt protease inhibitor cocktail. The prepared lysate was then mixed with the bead-bound biotinylated Oip5-as1. Following incubation, the proteins adhered to Oip5-as1 were eluted for further analysis using silver staining, mass spectrometry, and Western blotting techniques. Silver staining for protein visualization was performed using a Silver Staining Kit (Beyotime). Mass spectrometry for protein identification was conducted with a Q Exactive Orbitrap Mass Spectrometer (Thermo Scientific). In Western blot applications, proteins were transferred onto a polyvinylidene fluoride membrane (PVDF; Merck Millipore, Burlington, MA, USA) and detected with an Immobilon Western Chemiluminescent HRP Substrate (Merck Millipore).

To validate the specificity of the interaction between Oip5-as1 and AKAP1, competition assays with both biotinylated and unlabeled Oip5-as1 were performed. The unlabeled Oip5-as1 was synthesized in vitro using the T7 RNA polymerase, with biotin-UTP omitted from the reaction. HL-1 cell lysates were incubated with biotinylated Oip5-as1 bound to streptavidin-coated magnetic beads. This incubation was conducted under various conditions, either without or with unlabeled Oip5-as1 at concentrations of 0, 1, 5, and 15 times that of the biotinylated Oip5-as1. After incubation, the beads were isolated, and the bound proteins were analyzed via Western blotting for the presence of AKAP1. A decrease in the AKAP1 signal upon the addition of unlabeled Oip5-as1 would indicate a specific interaction between Oip5-as1 and AKAP1.

Upon determining the binding partners of Oip5-as1, it was segmented into three distinct parts to further clarify the specific sequence involved in the interaction via truncated RNA pull-down assays. The assays were performed in the same manner as with the full-length Oip5-as1. The captured complexes were analyzed by Western blotting.

### RNA immunoprecipitation assay

To investigate the interactions between Oip5-as1 and proteins, RNA immunoprecipitation (RIP) assays were carried out using an EZ-Magna RIP RNA-Binding Protein Immunoprecipitation Kit (Merck Millipore). HL-1 cell lysates was prepared with the supplied RIP lysis buffer, enriched with the Halt protease inhibitor cocktail and RNase inhibitor. The lysate was subsequently incubated with protein A/G magnetic beads that were conjugated with 5 µg of specific antibodies (anti-rabbit-IgG, AKAP1, or CaN; Cell Signaling Technology). Following an overnight incubation at 4 °C with gentle rotation, the RNA–protein complexes bound to the beads were washed with the supplied washing buffers. The complexes were then exposed to proteinase K to degrade the proteins, and the accompanying RNA was purified as per the manufacturer’s guidelines. The purified RNA was subsequently subjected to downstream RT-qPCR analysis to quantify the enrichment of Oip5-as1. Additionally, the EZ-Magna RIP Kit contains antibodies against SNRNP70 (small nuclear ribonucleoprotein 70 kDa) and primers for U1, which serve as positive controls for the experiments.

### Co-immunoprecipitation assays

Co-immunoprecipitation (Co-IP) assays were conducted utilizing a Pierce Crosslink Magnetic IP/Co-IP Kit (Thermo Scientific) according to the manufacturer’s instructions. In brief, 5 µg of specific antibodies (anti-rabbit-IgG, AKAP1, or CaN) were crosslinked to Protein A/G Magnetic Beads using the supplied crosslinking reagent. Cell lysis was accomplished using the provided IP Lysis/Wash Buffer augmented with the Halt protease inhibitor cocktail and phenylmethylsulfonyl fluoride (PMSF; Solarbio). The resulting cell lysate was incubated with the bead-bound antibodies to allow for the binding of the target protein. After the binding process, the beads were washed thrice with the IP Lysis/Wash Buffer to remove non-specifically bound proteins. The target protein, together with any interacting proteins, were then eluted from the beads using the provided Elution Buffer. The eluted proteins were subject to Western blot analysis to identify any co-immunoprecipitated proteins.

### Animals and treatments

Adult C57BL/6 mice, aged between 10 and 12 weeks and weighing between 20 and 25g, were procured from the Experimental Animal Center at Lanzhou University. These mice were maintained in a temperature-regulated environment (22 ± 2 ℃) with a 12-h light/dark cycle, and given unrestricted access to standard rodent chow and sterile water. All procedures involving the animals were approved by the Animal Care Committee at The First Hospital of Lanzhou University, and were executed in compliance with the National Institutes of Health’s guide for the care and use of laboratory animals.

A MI/R injury model was established by ligating the left anterior descending (LAD) coronary artery in mice. Initially, anesthesia was induced using 2% isoflurane, delivered via a small animal anesthesia machine (RWD Life Science, Shenzhen, Guangdong, China). Following endotracheal intubation, the animals were placed on a heating pad to maintain a consistent body temperature of 37 ± 0.5 ℃. Ventilation was provided using a rodent ventilator (Harvard Apparatus, Holliston, MA, USA), with continuous electrocardiogram (ECG) monitoring throughout the procedure. A left thoracotomy was conducted at the fourth intercostal space to expose the heart. The LAD artery was then ligated with 8-0 silk sutures for 30 min to induce ischemia, which was confirmed by an ST-segment elevation on the ECG. After the ischemic period, the ligature was released to allow reperfusion, indicated by the return of a red hue to the previously pale myocardium. The thoracic cavity was subsequently closed, and the animals were allowed to recover. For the sham-operated control animals, all surgical procedures were identical except for the LAD artery ligation. When required, the Mdivi-1 was administered through intravenous injection at a dosage of 1.2 mg/kg prior to the initiation of myocardial ischemia.

### Generation of Oip5-as1 conditional knockout mice

Conditional knockout mice for Oip5-as1 were generated using the CRISPR/Cas9 genome editing technology. Guide RNAs (gRNAs) targeting Oip5-as1 were created based on designs from the CRISPOR tool (http://crispor.tefor.net/). Cas9 mRNA, gRNAs, and donor vectors with loxP sites were co-injected into zygotes obtained from C57BL/6 female mice (Cyagen Biosciences, Suzhou, Jiangsu, China). PCR screening for loxP sites in positive F0 mice was conducted with a MiniBEST Universal Genomic DNA Extraction Kit (TaKaRa Biomedical Technology, Beijing, China) and a Taq Master Mix (Vazyme, Nanjing, Jiangsu, China), and the results were confirmed by Sanger sequencing. F1 offspring were bred by mating positive F0 mice with wild-type mice. The resulting F1 mice were then crossbred with Myh6-Cre mice (Cyagen Biosciences), leading to cardiomyocyte-specific recombination at the loxP sites and the generation of F2 offspring. Finally, the presence of Cre recombinase and the successful deletion of Oip5-as1 in the cardiac tissue were confirmed through genotyping of the F2 offspring. PCR primer sequences are provided in Additional file 1: Table S1.

### AAV9 vector production and administration

Overexpression of Oip5-as1 in mice was achieved using recombinant adeno-associated virus serotype 9 (AAV9) vectors coupled with a cardiac troponin T (cTNT) promoter. Constructs for Oip5-as1 overexpression (AAV-Oip5-as1) and a negative control (AAV9-empty viral particles, AAV-NC) were obtained from Genechem. Mice were delivered 1 × 10^12^ vg/mL AAV9 vector via intramyocardial injection using a microsyringe (Hamilton Company, Reno, NV, USA), in accordance with the established experimental protocol [22]. RT-qPCR was utilized to confirm the effectiveness of myocardial Oip5-as1 overexpression.

### Echocardiographic assessment

Echocardiographic assessments were conducted using a Vevo 3100 Imaging System (Fujifilm VisualSonics, Toronto, Canada) equipped with a high-frequency linear array transducer. Anesthesia was initiated in mice with 2% isoflurane and maintained between 0.8 and 1.5% via a nose cone. Subsequently, M-mode echocardiographic images were captured in a parasternal long-axis orientation at the level of the papillary muscles. Parameters such as the left ventricular ejection fraction (LVEF) and left ventricular fractional shortening (LVFS) were computed to assess cardiac function. All evaluations were undertaken by an investigator unaware of the experimental group assignments. For accuracy and reproducibility, measurements averaged at least three cardiac cycles. Data were recorded and analyzed using a Vevo LAB analysis software (Fujifilm VisualSonics).

### Infarct size determination

Infarct size was determined by triphenyltetrazolium chloride (TTC) staining. At 24 h following MI/R injury, hearts were promptly excised and immersed in ice-cold saline. Subsequently, each heart was sectioned into six slices and immediately plunged into a 2% TTC solution (Solarbio), followed by an incubation period of 20 min at 37 °C. The TTC staining process resulted in the viable myocardium appearing red, while the infarcted tissue remained unstained, thus appearing pale. Digital captures of the slices were taken for analysis using the ImageJ software. Infarct size was expressed as a percentage of the total ventricular area, computed by aggregating the area of infarction across all slices and normalizing by the total ventricular area of the slices.

### TUNEL assay

Apoptosis in myocardial tissues was evaluated using terminal deoxynucleotidyl transferase dUTP nick end labeling (TUNEL) assay, following the instructions provided with a TUNEL Apoptosis Detection Kit (Beyotime). The myocardial tissue sections, embedded in paraffin, were subjected to deparaffinization, rehydration, antigen retrieval, and endogenous peroxide deactivation. The sections were then incubated with the TUNEL reaction mixture for an hour at 37 °C. Post-incubation, they were labeled with Streptavidin-HRP working solution and developed using DAB. Hematoxylin was used for counterstaining. Apoptotic cells were identified by brown staining and classified as TUNEL-positive. An investigator, blind to the treatment conditions, utilized the Olympus microscope for image capture and the ImageJ software for quantifying the percentage of TUNEL-positive cells.

### Hematoxylin and eosin staining

Histopathological alterations in myocardial tissue sections were observed using hematoxylin and eosin (H&E) staining. The procedure followed the protocol provided by an H&E Staining Kit (Beyotime).

### Transmission electron microscopy

Mitochondrial morphology in myocardial tissues was assessed using transmission electron microscopy (TEM). The tissues were initially fixed in a 3% glutaraldehyde solution and subsequently post-fixed in 1% osmium tetroxide (Sigma-Aldrich). The samples were then dehydrated in an ascending acetone series and embedded in Epon 812 (SPI Supplies, West Chester, PA, USA). Ultrathin sections, with thicknesses ranging from 60–90 nm, were sliced with a diamond knife and stained with uranyl acetate (SPI Supplies) and lead citrate (Electron Microscopy Sciences, Hatfield, PA, USA). Images of interfibrillar mitochondria were captured at a magnification of 10,000 × using a JEM-1400-FLASH Transmission Electron Microscope (JOEL, Tokyo, Japan). An investigator, blinded to the experimental conditions, quantified various mitochondrial morphological parameters, such as area, perimeter, and circularity, using the ImageJ software [31].

### Lactate dehydrogenase detection

The activity of lactate dehydrogenase (LDH) in mouse serum was assessed using an LDH Assay Kit (Solarbio), following the manufacturer’s instructions. The OD was measured at a wavelength of 450 nm with the microplate reader. The LDH activity was determined by comparing the OD values of the test samples with a standard curve generated from known LDH concentrations.

### RNA isolation and RT-qPCR

Total RNA was extracted from the samples using a TRIzol Reagent (Invitrogen, Carlsbad, CA, USA), following the manufacturer’s instructions. The isolated RNA was reverse-transcribed into cDNA using a HiScript III 1st Strand cDNA Synthesis Kit (Vazyme). Subsequent qPCR was performed using a ChamQ Universal SYBR qPCR Master Mix (Vazyme), adhering to the recommended protocol. RT-qPCR was executed on a QuantStudio 5 Real-Time PCR System (Thermo Fisher Scientific). Relative gene expression levels were quantified using the 2^−ΔΔCT^ method, with normalization against the housekeeping gene Actb. Primer sequences, furnished by Sangon Biotech (Shanghai, China), are listed in Additional file 1: Table S1.

### Western blot analysis

Cell or tissue lysates were prepared with the RIPA buffer supplemented with the protease inhibitor cocktail, PMSF, and a phosphatase inhibitor cocktail (Solarbio). Protein concentration was quantified with a BCA Protein Assay Kit (Solarbio). Proteins were separated via 10%-15% SDS-PAGE, then transferred to the PVDF membrane, and blocked with 5% non-fat milk or bovine serum albumin for 2 h. The blocked membranes were incubated overnight at 4 °C with primary antibodies (Additional file 1: Table S2). Detection of primary antibodies was carried out using HRP-conjugated secondary antibodies, either anti-rabbit or anti-mouse (ZSGB-BIO, Beijing, China), and visualized with the Immobilon kit. Gel images were quantified using the ImageJ software.

### Statistical analysis

Quantitative data are represented as mean ± standard deviation (SD). Comparisons between two groups were undertaken using an unpaired Student’s *t*-test, while for multiple groups, analysis was performed using a one-way analysis of variance (ANOVA) followed by Tukey’s post hoc test. A *P*-value of less than 0.05 is considered statistically significant. All statistical analyses were conducted using GraphPad Prism 8.00 software (GraphPad Software, San Diego, CA, USA).

## Results

### Oip5-as1 attenuates H/R-induced damage in HL-1 cells

**Fig. 1:**
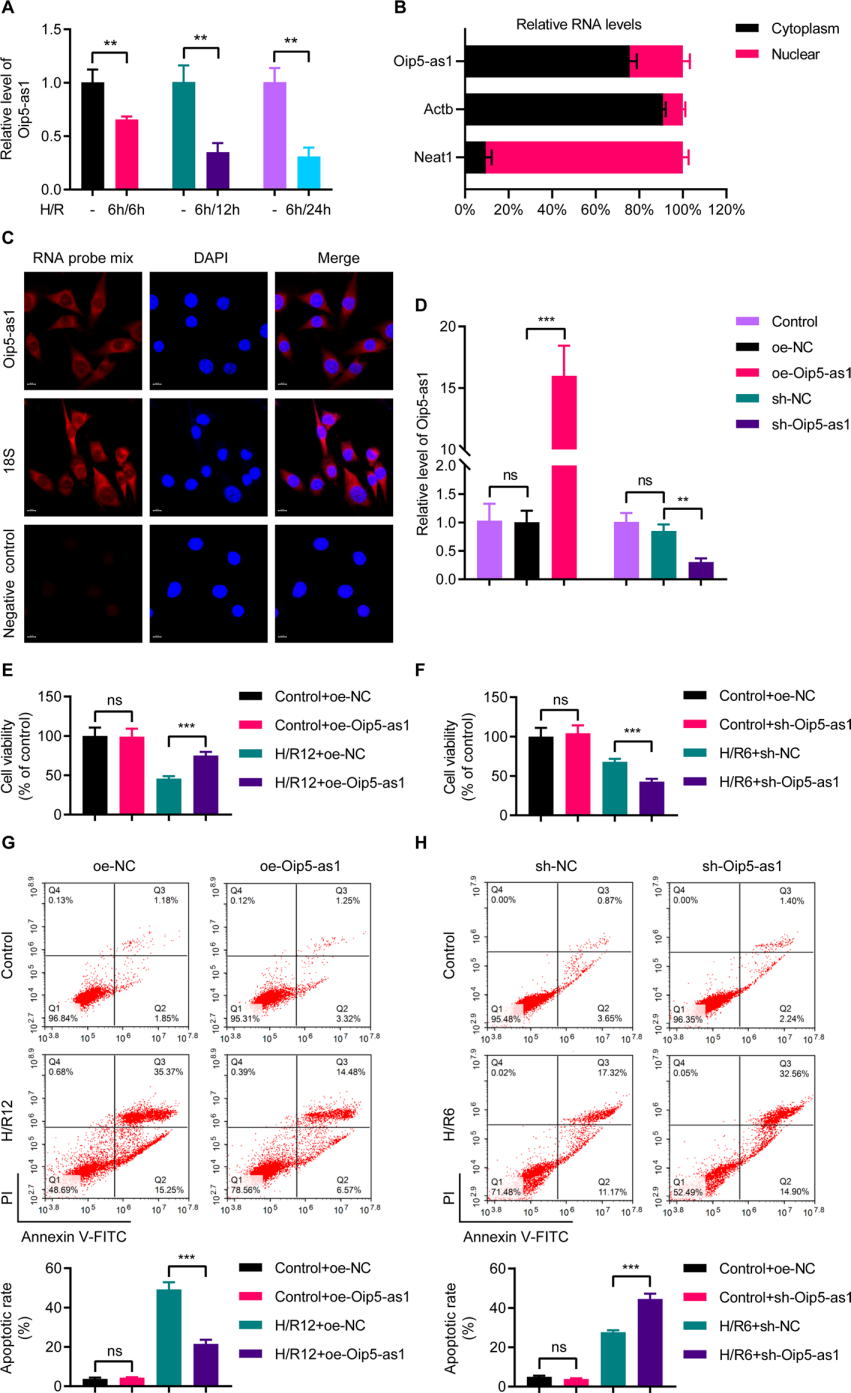
The role of Oip5-as1 in mitigating H/R-induced damage in HL-1 cells. **A** RT-qPCR analysis of Oip5-as1 expression levels in HL-1 cells subjected to different durations of hypoxia/reoxygenation (H/R). **B** Nuclear and cytoplasmic fractionation followed by RT-qPCR analysis of Oip5-as1 expression in HL-1 cells. Actb is utilized as a cytoplasmic reference gene, whereas lncRNA Neat1 is employed as a nuclear reference gene. **C** FISH experiment showing the cytoplasmic localization of Oip5-as1 in HL-1 cells. The 18S probe is used as a positive control to confirm the specificity of the hybridization, and the scramble sequence probe is employed as a negative control to ensure that non-specific hybridization is absent. Scale bar, 10 μm. **D** RT-qPCR analysis of Oip5-as1 expression levels in untransfected HL-1 cardiomyocytes (Control) and cells transfected with lentiviral vectors for Oip5-as1 overexpression or knockdown. **E** CCK-8 assay results indicating increased viability in HL-1 cells due to Oip5-as1 overexpression under hypoxic conditions for 6 h followed by 12 h of reoxygenation (H/R12). **F** CCK-8 assay results showing decreased viability in HL-1 cells following Oip5-as1 knockdown under hypoxic conditions for 6 h followed by 6 h of reoxygenation (H/R6). **G** Flow cytometry analysis of HL-1 cells stained with Annexin V-FITC/PI, illustrating the reduction in cell apoptosis due to Oip5-as1 overexpression under H/R12 conditions. **H** Flow cytometry analysis of HL-1 cells stained with Annexin V-FITC/PI, indicating the increase in cell apoptosis following Oip5-as1 knockdown under H/R6 conditions. Data are presented as mean ± standard deviation, *n* = 3. **P < 0.01 and ***P < 0.001. *ns* nonstatistically significant, *RT-qPCR* real-time quantitative polymerase chain reaction, *Actb* actin beta, *Neat1* nuclear paraspeckle assembly transcript 1, *FISH* fluorescence in situ hybridization, *DAPI* 4', 6-diamidino-2-phenylindole, *FITC* fluorescein lsothiocyanate, *PI* propidium iodide, *oe-Oip5-as1* the overexpressed lentivirus for Oip5-as1, *oe-NC* the overexpressed lentivirus for negative control, *sh-Oip5-as1* the short hairpin RNA (shRNA)-mediated lentivirus targeting Oip5-as1, *sh-NC* the negative control shRNA lentivirus

To elucidate the role of Oip5-as1 in MI/R injury, we initially utilized RT-qPCR to examine how varying H/R durations affect the expression levels of Oip5-as1 in HL-1 cells. The data revealed a decreasing trend in Oip5-as1 expression levels subjected to consistent hypoxia duration but varying reoxygenation times (Fig. 1A). A 6-h hypoxia condition followed by 12 h of reoxygenation resulted in a decrease in Oip5-as1 levels to approximately 0.4-fold compared to normal conditions (Fig. 1A).

The functions of lncRNAs are closely associated with their subcellular localization. To determine the cellular localization of Oip5-as1, we employed nuclear-cytoplasmic separation RT-qPCR. The RT-qPCR results indicated that Oip5-as1 is primarily located in the cytoplasm (Fig. 1B). We further validated this cytoplasmic localization finding through the FISH assay, which confirmed the predominant cytoplasmic localization of Oip5-as1 (Fig. 1C).

To further explore the biological role of Oip5-as1, we utilized the lentiviral transfection method for both gain-of-function and loss-of-function assays. For Oip5-as1 overexpression in HL-1 cells, RT-qPCR analysis demonstrated a 16.0-fold augmentation in Oip5-as1 expression in the oe-Oip5-as1 group relative to the oe-NC group (Fig. 1D). Conversely, for Oip5-as1 knockdown, the RT-qPCR data indicated a reduction in Oip5-as1 expression to about 0.3-fold in the sh-Oip5-as1 group when compared with the sh-NC group (Fig. 1D). In order to effectively observe the influence of Oip5-as1 on HL-1 cells under H/R conditions, we implemented a scenario of 6 h of hypoxia followed by 12 h of reoxygenation (H/R12) during the overexpression experiment. On the other hand, for the knockdown experiment, we applied a condition involving 6 h of hypoxia followed by 6 h of reoxygenation (H/R6).

In the CCK-8 assay, cell viability was unaffected by either the oe-NC or oe-Oip5-as1 groups (Fig. 1E). However, under H/R12 conditions, the oe-Oip5-as1 group demonstrated a significant increase in cell viability compared to oe-NC (Fig. 1E). Conversely, the sh-NC and sh-Oip5-as1 groups showed no significant difference in cell viability under normal conditions (Fig. 1F), although the sh-Oip5-as1 group experienced a notable reduction in cell viability under H/R6 conditions (Fig. 1F).

Flow cytometry experiments revealed that cell apoptosis remained unchanged in both the oe-NC and oe-Oip5-as1 groups (Fig. 1G). Under H/R12 conditions, cell apoptosis in the oe-Oip5-as1 group decreased compared to oe-NC (Fig. 1G). In contrast, the sh-Oip5-as1 group exhibited a significant increase in cell apoptosis compared to sh-NC under the H/R6 conditions (Fig. 1H). These results suggest that Oip5-as1 plays a protective role against H/R-induced injury.

### Oip5-as1 inhibits excessive mitochondrial fission triggered by H/R in HL-1 cells

**Fig. 2:**
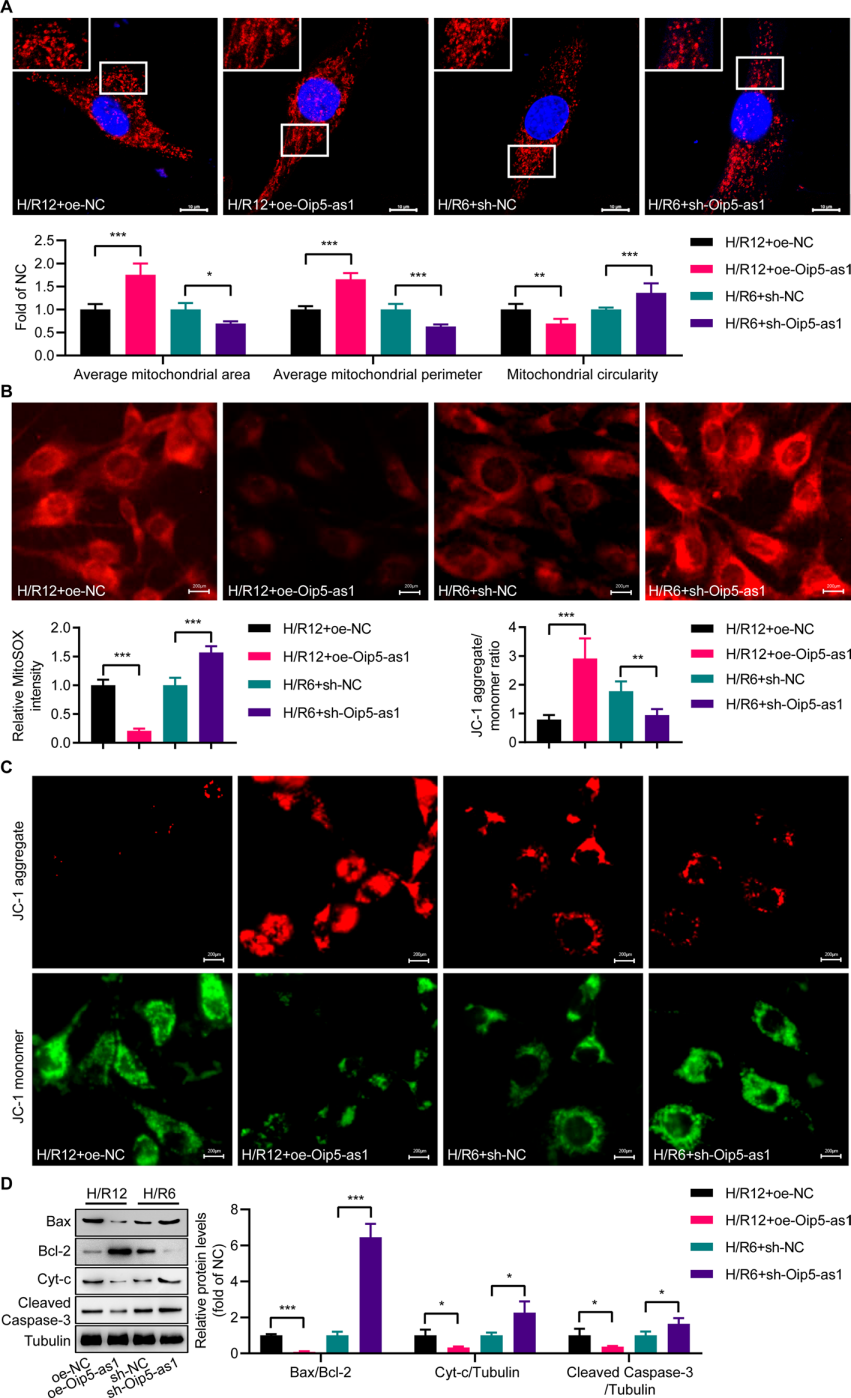
Oip5-as1 attenuates H/R-induced mitochondrial fission in HL-1 cells. **A** Mitochondrial morphology analysis shows that overexpression of Oip5-as1 increases mitochondrial area and perimeter and decreased circularity, while Oip5-as1 knockdown decreases area and perimeter and increases circularity in HL-1 cells subjected to H/R compared to their respective negative controls. The mitochondria are visualized using an antibody specific to Tom-20, while the cell nuclei are stained with DAPI. The upper panels show enlarged views of the regions indicated by the boxes in the lower panels. Scale bar, 10 μm. **B** MitoSOX staining reveals that oe-Oip5-as1 reduced while sh-Oip5-as1 increased mitochondrial ROS levels in comparison to their respective controls. Scale bar, 200 μm. **C** JC-1 assay demonstrates that oe-Oip5-as1 maintains while sh-Oip5-as1 decreases mitochondrial membrane potential relative to their respective controls. Scale bar, 200 μm. **D** Western blots and quantitative analysis indicate that oe-Oip5-as1 suppresses while sh-Oip5-as1 upregulates levels of mitochondrial pro-apoptotic proteins including Bax/Bcl-2, Cyt-c, and Cleaved Caspase-3 compared to their respective controls. For the Oip5-as1 overexpression experiment, a hypoxia/reoxygenation (H/R) protocol consisting of 6 h of hypoxia followed by 12 h of reoxygenation (H/R12) was implemented. Conversely, in the Oip5-as1 knockdown experiment, HL-1 cells were subjected to an H/R protocol of 6 h of hypoxia followed by 6 h of reoxygenation (H/R6). Data are presented as mean ± standard deviation, *n* = 3. *P < 0.05, **P < 0.01, and ***P < 0.001. Tom-20, translocase of outer mitochondria 20. *DAPI* 4', 6-diamidino-2-phenylindole. *ROS* reactive oxygen species, *Bax* Bcl-2 associated X protein, *Bcl-2* B-cell lymphoma 2, *Cyt-c* cytochrome c, *oe-Oip5-as1* the overexpressed lentivirus for Oip5-as1, *oe-NC* the overexpressed lentivirus for negative control, *sh-Oip5-as1* the short hairpin RNA (shRNA)-mediated lentivirus targeting Oip5-as1, *sh-NC* the negative control shRNA lentivirus

Mitochondrial fission has been established as a significant contributor to H/R-induced apoptotic damage in cardiomyocytes. In the subsequent research, we continue to explore the potential regulatory role of Oip5-as1 in mitochondrial fission. Evaluations of mitochondrial morphology in normal HL-1 cells disclosed no notable differences in average mitochondrial area, perimeter, and circularity among the oe-Oip5-as1, sh-Oip5-as1, oe-NC, and sh-NC groups (Additional file 2: Fig. S1A). In HL-1 cells treated with H/R12, transfection with oe-Oip5-as1 significantly increased the average mitochondrial area and perimeter, and decreased circularity in comparison to transfection with oe-NC (Fig. 2A). In contrast to sh-NC transfection, sh-Oip5-as1 transfection significantly reduced the average mitochondrial area and perimeter but increased circularity in HL-1 cells under H/R6 condition (Fig. 2A).

Excessive mitochondrial fission can lead to mitochondrial dysfunction, thereby inflicting damage upon cardiomyocytes. MitoSOX staining showed no change in fluorescence intensity in normal HL-1 cells with oe-Oip5-as1, sh-Oip5-as1, oe-NC or sh-NC transfection (Additional file 2: Fig. S1B). Under H/R induction, oe-Oip5-as1 attenuated while sh-Oip5-as1 amplified the fluorescence intensity (Fig. 2B). Similarly, JC-1 assay indicated no difference in mitochondrial fluorescence of normal HL-1 cells transfected with oe-Oip5-as1, sh-Oip5-as1, oe-NC or sh-NC (Additional file 2: Fig. S1C). However, upon H/R induction, cells with oe-Oip5-as1 had higher aggregate/monomer fluorescence ratio compared to oe-NC cells, whereas cells with sh-Oip5-as1 displayed further decrease in the ratio versus sh-NC cells (Fig. 2C). Western blot analysis showed the H/R-induced increase in Bax/Bcl-2, Cyt-c, and Cleaved-Caspase-3 was significantly attenuated in oe-Oip5-as1 transfected HL-1 cells, while substantially enhanced in sh-Oip5-as1 transfected cells, respectively (Fig. 2D). These findings suggest that Oip5-as1 limits excessive mitochondrial fission and preserves mitochondrial function in cells subjected to H/R stress.

### Oip5-as1 enhances phosphorylation of DRP1 at Ser637 site

**Fig. 3:**
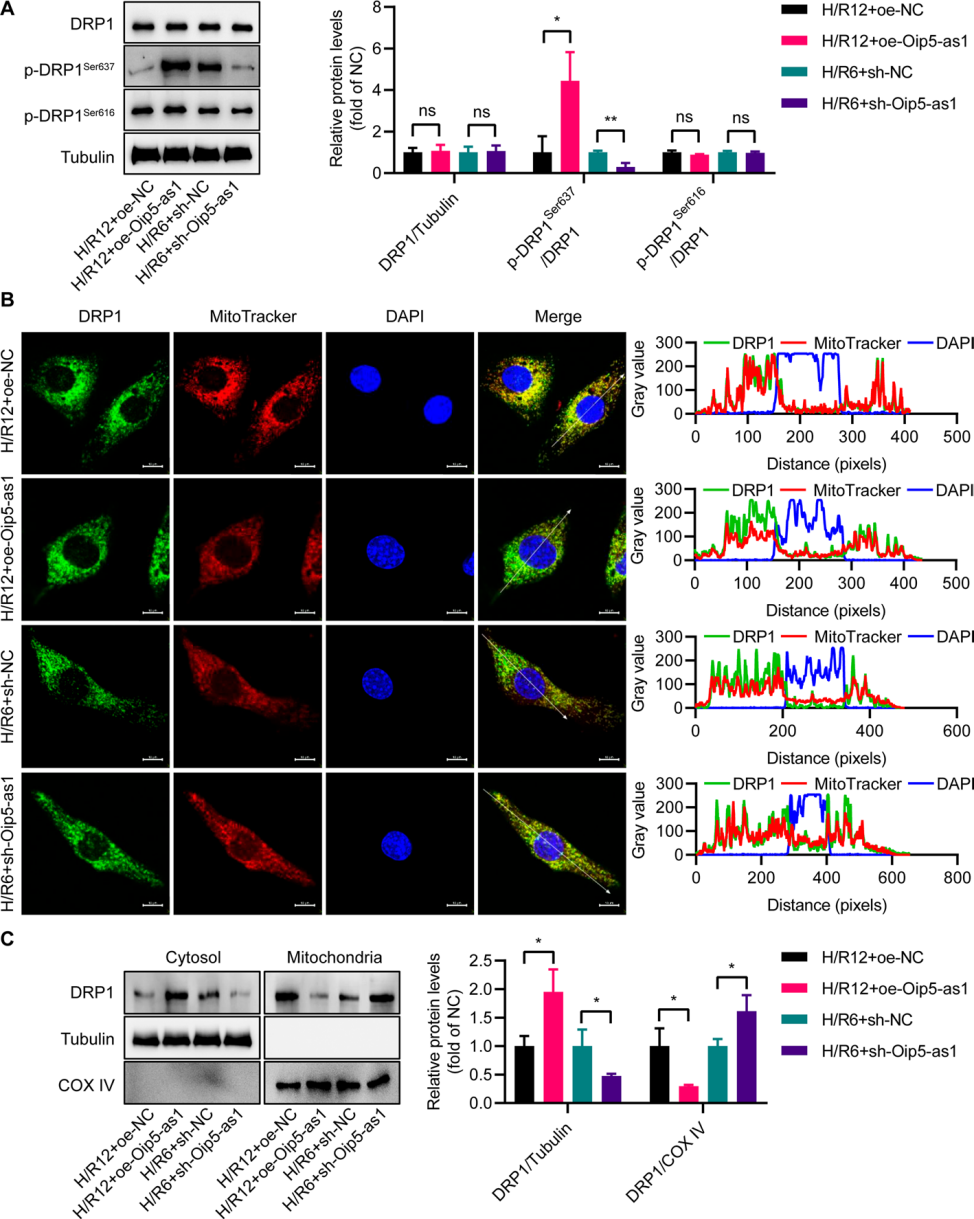
Oip5-as1 inhibits DRP1 mitochondrial translocation by promoting DRP1 phosphorylation at Ser637 under H/R conditions. **A** Western blot and quantitative analyses are used to detect the expression levels of DRP1 and DRP1 phosphorylation at the Ser637 or Ser616 position (p-DRP1^Ser637^ or p-DRP1^Ser616^). **B** Representative immunofluorescence images and co-localization analysis of DRP1 in HL-1 cells under H/R conditions, showing DRP1 stained with the antibody in green, mitochondria marked with MitoTracker in red, and nuclei stained with DAPI in blue. Scale bar, 10 μm. **C** Western blots and quantitative analysis of the levels of DRP1 in cytoplasmic and mitochondrial fractions from HL-1 cells transfected with oe-Oip5-as1, sh-Oip5-as1, oe-NC, and sh-NC after H/R treatment. COX IV protein is utilized as a mitochondrial marker to identify mitochondrial fractions, whereas Tubulin protein is employed as a cytoplasmic marker for cytoplasmic fractions. The Oip5-as1 overexpression experiment employed an H/R12 protocol (6 h of hypoxia followed by 12 h of reoxygenation), whereas the Oip5-as1 knockdown experiment utilized an H/R6 protocol (6 h of hypoxia followed by 6 h of reoxygenation). Data are presented as mean ± standard deviation, *n* = 3. *P < 0.05 and **P < 0.01. *ns* nonstatistically significant, *H/R* hypoxia/reoxygenation, *DRP1* dynamin-related protein 1, *COX IV* cytochrome c oxidase subunit IV, *DAPI* 4', 6-diamidino-2-phenylindole, *oe-Oip5-as1* the overexpressed lentivirus for Oip5-as1, *oe-NC* the overexpressed lentivirus for negative control, *sh-Oip5-as1* the short hairpin RNA (shRNA)-mediated lentivirus targeting Oip5-as1, *sh-NC* the negative control shRNA lentivirus

To elucidate the mechanism of action of Oip5-as1, we firstly employed Western blot techniques to assess the effect of Oip5-as1 on the expression of proteins mediating mitochondrial fission. The results indicated that the overexpression or knockdown of Oip5-as1 did not lead to significant alterations to the expression levels of total DRP1 protein (Fig. 3A). Considering that the post-translational modifications of the DRP1 protein play a crucial role in influencing mitochondrial fission, we examined the alterations in phosphorylation at the Ser616 and Ser637 sites of DRP1. Western blot analysis demonstrated an increased level of DRP1 phosphorylation at the Ser637 position (p-DRP1^Ser637^) upon transfection with oe-Oip5-as1, conversely, a decreased level was observed with sh-Oip5-as1 transfection (Fig. 3A). However, no changes were detected at the DRP1^Ser616^ phosphorylation levels, regardless of transfection with either oe-Oip5-as1 or sh-Oip5-as1 (Fig. 3A).

Subsequent investigation was undertaken to ascertain whether Oip5-as1 modulates the mitochondrial localization of DRP1 by enhancing the level of p-DRP1^Ser637^. Immunofluorescence co-localization experiments demonstrated that oe-Oip5-as1 transfection led to a notable suppression in the translocation of DRP1 from the cytosol to the mitochondria compared to the controls (Fig. 3B). In contrast, sh-Oip5-as1 notably enhanced DRP1’s mitochondrial translocation in HL-1 cells subjected to H/R6 injury when compared to the controls (Fig. 3B). Western blot assays indicated that following H/R treatment, HL-1 cells transfected with oe-Oip5-as1 exhibited a significant reduction in mitochondrial DRP1 expression when compared to the oe-NC transfected cells (Fig. 3C). Conversely, the sh-Oip5-as1 transfected cells displayed a significant increase in mitochondrial DRP1 expression relative to the cells transfected with sh-NC (Fig. 3C). These results demonstrate that Oip5-as1 enhances p-DRP1^Ser637^ levels, which in turn inhibits DRP1’s translocation to mitochondria.

### p-DRP1^Ser637^ mediates the effect of Oip5-as1 on mitochondrial fission

**Fig. 4:**
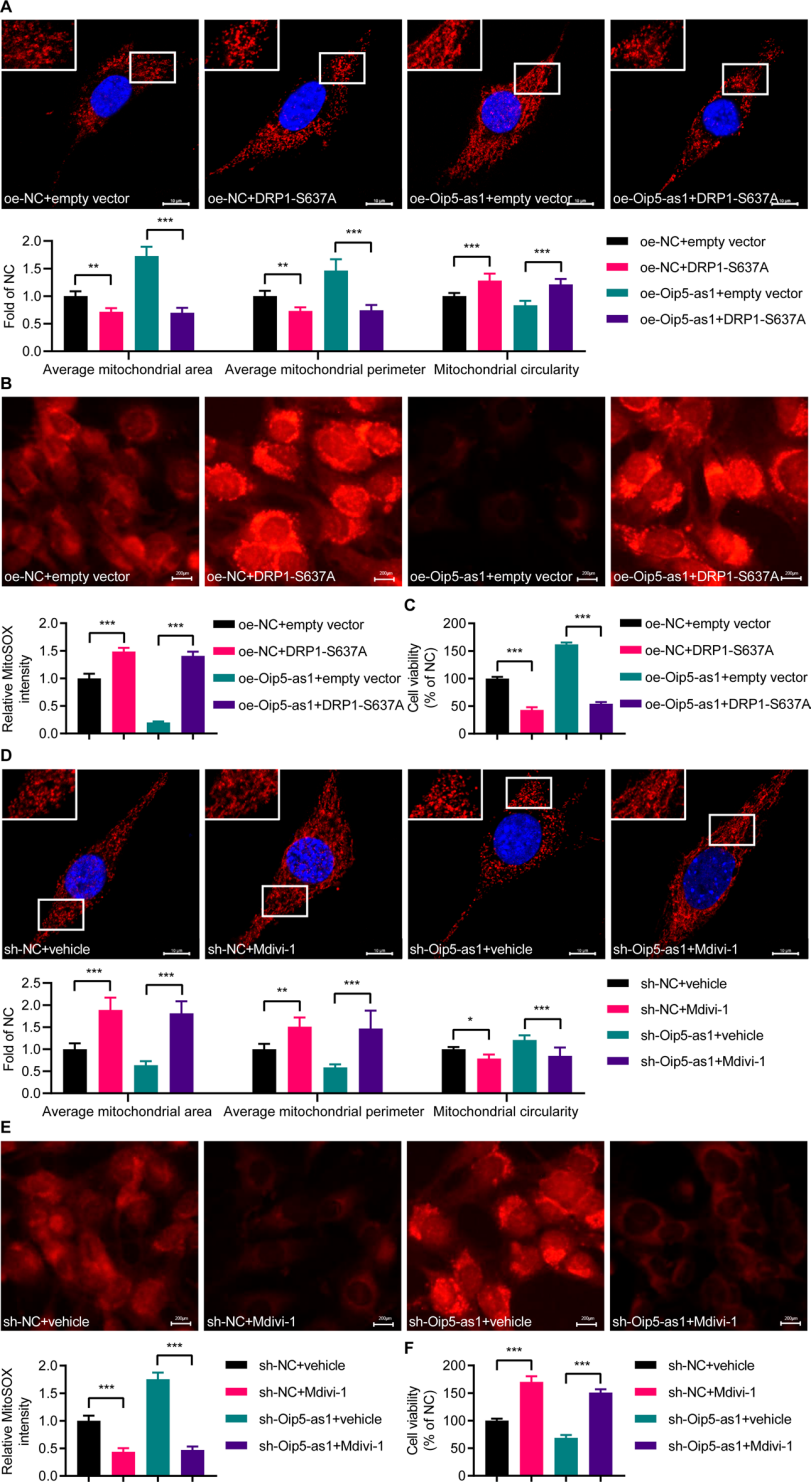
The role of Oip5-as1 in inhibiting H/R-induced mitochondrial fission is dependent on DRP1 phosphorylation at Ser637. **A** Mitochondrial morphology analysis shows that transfection of phospho-deficient DRP1-S637A plasmid in HL-1 cells overexpressing Oip5-as1 and subjected to H/R induction leads to a decrease in average mitochondrial area and perimeter, accompanied by an increase in circularity levels. The mitochondria are visualized using an antibody specific to Tom-20, while the cell nuclei are stained with DAPI. The upper panels show enlarged views of the regions indicated by the boxes in the lower panels. Scale bar, 10 μm. **B** MitoSOX staining demonstrates an elevation in mitochondrial ROS levels in HL-1 cells overexpressing Oip5-as1 and undergoing H/R induction upon transfection with the DRP1-S637A plasmid. Scale bar, 200 μm. **C** CCK-8 assay shows a reduction in cell viability in HL-1 cells overexpressing Oip5-as1 and subjected to H/R induction following transfection with the DRP1-S637A plasmid. **D** Mitochondrial morphology analysis reveals that the administration of Mdivi-1 in HL-1 cells with Oip5-as1 knockdown and subjected to H/R induction leads to an increase in average mitochondrial area and perimeter, accompanied by a decrease in circularity levels. The mitochondria are visualized using an antibody specific to Tom-20, while the cell nuclei are stained with DAPI. The upper panels show enlarged views of the regions indicated by the boxes in the lower panels. Scale bar, 10 μm. **E** MitoSOX staining indicates a reduction in mitochondrial ROS levels in HL-1 cells with Oip5-as1 knockdown and undergoing H/R induction upon use of Mdivi-1. Scale bar, 200 μm. **F** CCK-8 assay demonstrates an elevation in cell viability in HL-1 cells with Oip5-as1 knockdown and subjected to H/R induction following Mdivi-1 treatment. For the Oip5-as1 overexpression experiment, an H/R12 protocol (6 h of hypoxia followed by 12 h of reoxygenation) was employed, whereas an H/R6 protocol (6 h of hypoxia followed by 6 h of reoxygenation) was utilized in the Oip5-as1 knockdown experiment. Data are presented as mean ± standard deviation, *n* = 3. *P < 0.05, **P < 0.01, and ***P < 0.001. *ns* nonstatistically significant, *H/R* hypoxia/reoxygenation, *Mdivi-1* mitochondrial division inhibitor-1 (an inhibitor of DRP1), *Tom-20* translocase of outer mitochondria 20, *DAPI* 4', 6-diamidino-2-phenylindole, *ROS* reactive oxygen species, *oe-Oip5-as1* the overexpressed lentivirus for Oip5-as1, *oe-NC* the overexpressed lentivirus for negative control, *sh-Oip5-as1* the short hairpin RNA (shRNA)-mediated lentivirus targeting Oip5-as1, *sh-NC* the negative control shRNA lentivirus

We further examined whether the effect of Oip5-as1 on mitochondrial fission is dependent on the upregulation of p-DRP1^Ser637^ levels. Assessment of mitochondrial morphology indicated that in HL-1 cells overexpressing Oip5-as1 and undergoing H/R12 induction, transfection with the phosphorylation-inhibiting mutant plasmid, DRP1-S637A, resulted in a decrease in average mitochondrial area and perimeter, and an elevation in circularity levels, compared to the cells transfected with the empty vector plasmid (Fig. 4A). MitoSOX staining results showed that transfection with the DRP1-S637A plasmid significantly increased the fluorescence intensity compared to the empty vector plasmid in HL-1 cells overexpressing Oip5-as1 and subjected to H/R12 injury (Fig. 4B). Using the CCK-8 assay, we assessed cell viability and found a decrease in HL-1 cells overexpressing Oip5-as1 and subjected to H/R12 induction after transfection with the DRP1-S637A plasmid, compared to those transfected with the empty vector plasmid (Fig. 4C).

Additionally, the administration of Mdivi-1 (an inhibitor of DRP1) in HL-1 cells, which have undergone Oip5-as1 knockdown and H/R6 treatment, could augment the mitochondrial area and perimeter, diminish circularity (Fig. 4D), and subsequently decrease the fluorescence intensity detected in MitoSOX staining (Fig. 4E). This treatment also increased cell viability in the CCK-8 assay (Fig. 4F). Our findings propose that Oip5-as1 suppresses H/R-induced excessive mitochondrial fission by promoting the level of p-DRP1^Ser637^.

### Oip5-as1 specifically binds to AKAP1 protein

**Fig. 5:**
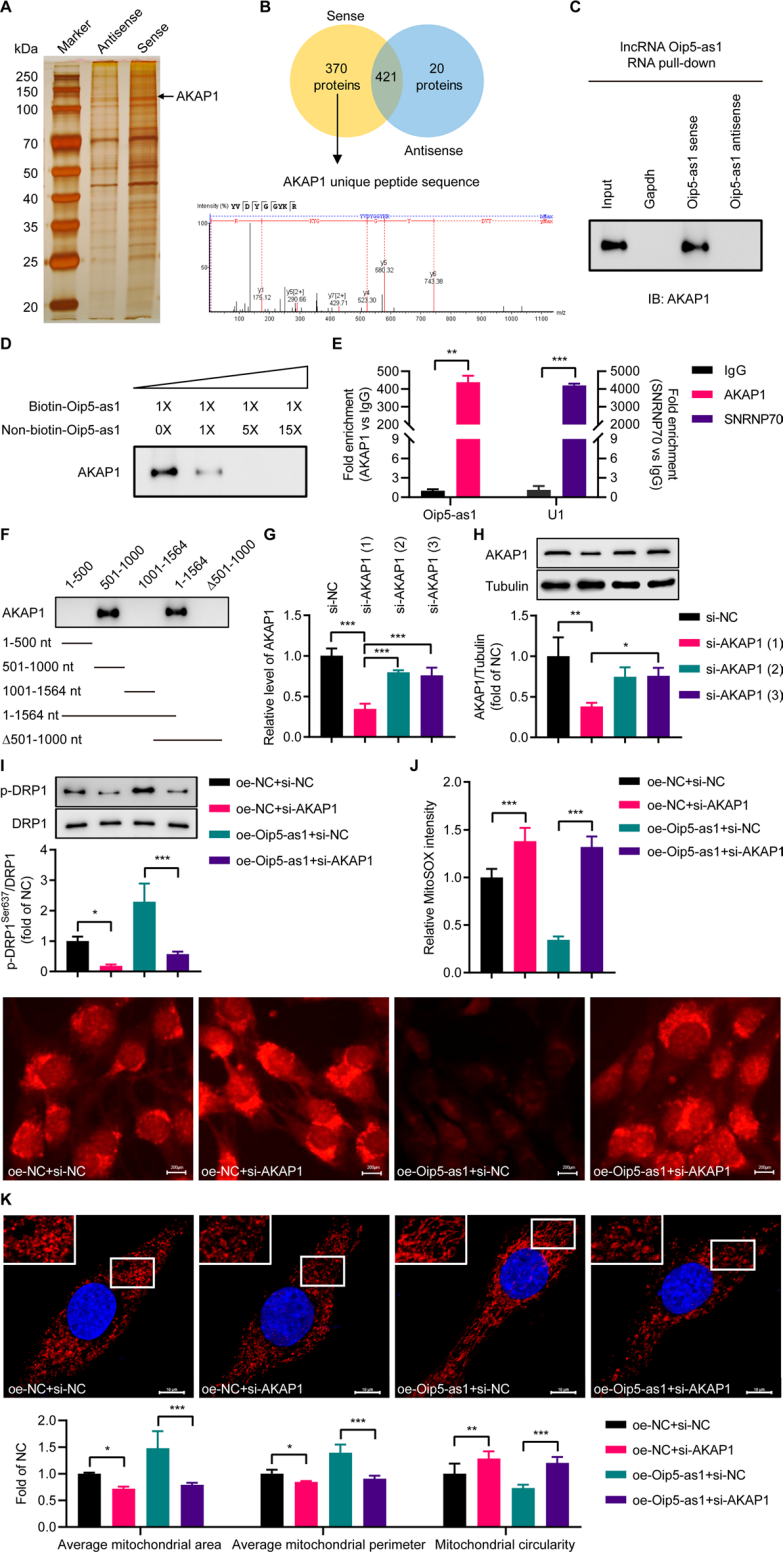
Interaction between Oip5-as1 and AKAP1 protein. **A** RNA pulldown assays are performed using biotinylated sense or antisense Oip5-as1. Proteins from HL-1 cell extracts are separated by SDS-PAGE and visualized by silver staining. A specific band corresponding to Oip5-as1, as indicated by the black arrow, is analyzed by mass spectrometry. **B** Venn diagram of distinct proteins binding to sense versus antisense Oip5-as1 in mass spectrometry analysis. AKAP1 is identified as an Oip5-as1-interacting protein. **C** Immunoblotting confirmation of AKAP1 binding specifically to Oip5-as1 sense RNA compared to antisense Oip5-as1 and Gapdh RNA. **D** Competition assay demonstrating concentration-dependent inhibition of biotinylated Oip5-as1 binding to AKAP1 by unlabeled Oip5-as1. **E** RNA immunoprecipitation showing endogenous Oip5-as1 retrieval by AKAP1 immunoprecipitation from HL-1 cell extracts versus IgG control, measured by RT-qPCR. Anti-SNRNP70 as positive control. **F** RNA pull-down assay using truncated Oip5-as1 variants to map Oip5-as1 regions interacting with AKAP1 by immunoblotting. **G** RT-qPCR analysis is performed to measure AKAP1 mRNA levels in HL-1 cells following transfection with either AKAP1-specific siRNAs (si-AKAP1) or negative control siRNA (si-NC). **H** Western blot and quantitative analyses are conducted to assess the levels of AKAP1 protein in HL-1 cells following treatment with si-AKAP1. **I** Western blot and quantification analyses reveal that si-AKAP1 reversed the increase in p-DRP1^Ser637^ levels induced by Oip5-as1 overexpression in HL-1 cells undergoing hypoxia/reoxygenation (H/R). **J** MitoSOX staining shows elevated mitochondrial ROS in Oip5-as1-overexpressing HL-1 cells treated with si-AKAP1 during H/R. Scale bar, 200 μm. **K** Mitochondrial morphology analysis indicates si-AKAP1 transfection leads to decreased mitochondrial area and perimeter, and correspondingly increased circularity in HL-1 cells overexpressing Oip5-as1 during H/R. The mitochondria are visualized using an antibody specific to Tom-20, while the cell nuclei are stained with DAPI. The upper panels show enlarged views of the regions indicated by the boxes in the lower panels. Scale bar, 10 μm. Data are presented as mean ± standard deviation, *n* = 3. *P < 0.05, **P < 0.01, and ***P < 0.001. *AKAP1* a kinase anchor protein 1, *Tom-20* translocase of outer mitochondria 20, *DAPI* 4', 6-diamidino-2-phenylindole, *ROS* reactive oxygen species, *RT-qPCR* real-time quantitative polymerase chain reaction, *oe-Oip5-as1* the overexpressed lentivirus for Oip5-as1, *oe-NC* the overexpressed lentivirus for negative control

lncRNAs are capable of regulating genes via specific RNA–protein interactions. To elucidate the mechanism underlying Oip5-as1’s promotion of DRP1 phosphorylation, RNA pulldown assays were utilized to detect proteins that bind to Oip5-as1. Silver staining and mass spectrometry analyses identified AKAP1 as a protein interacting with Oip5-as1 (Fig. 5A, B). This interaction was independently corroborated by Western blot analysis, which demonstrated the presence of AKAP1 in the Oip5-as1 protein complex, but not in complexes involving antisense Oip5-as1 or Gapdh (Fig. 5C).

To ascertain the specific interaction between AKAP1 and Oip5-as1, we conducted the competitive assay, which revealed that the association between AKAP1 and Oip5-as1 was competitively inhibited in a dose-dependent manner by the introduction of non-biotinylated Oip5-as1 (Fig. 5D). Moreover, the RIP experiment was undertaken to further substantiate the interaction between Oip5-as1 and AKAP1. As anticipated, a significant enrichment of Oip5-as1 was observed in the AKAP1-associated RIP, contrasting the RIP operated with Immunoglobulin G (IgG) as a control (Fig. 5E).

Additionally, we synthesized a series of truncated variants of Oip5-as1 to map the exact binding domain for AKAP1. Our findings indicated that the region spanning nucleotides from 501 to 1,000 within Oip5-as1 was capable of interacting with AKAP1 (Fig. 5F).

Numerous studies have established that AKAP1 plays a protective role in MI/R injury. We further explored whether AKAP1 mediates Oip5-as1-induced DRP1 phosphorylation and its associated inhibition of excessive mitochondrial fission. To silence AKAP1 expression in HL-1 cells, three siRNA sequences targeting AKAP1 were employed. The first sequence yielded the most significant reduction in both mRNA and protein levels and was therefore selected for subsequent experiments (Fig. 5G, H). Western blot analysis showed that AKAP1 silencing significantly reduced the protein levels of p-DRP1^Ser637^ in HL-1 cells with Oip5-as1 overexpression under H/R12 conditions (Fig. 5I). Using MitoSOX staining, we found an increase in mtROS levels in Oip5-as1-overexpressing HL-1 cells after si-AKAP1 intervention (Fig. 5J). Additionally, mitochondrial assessment revealed that si-AKAP1 significantly reduced mitochondrial area and perimeter, and increased their circularity in these cells (Fig. 5K). These results demonstrate that the interplay between Oip5-as1 and AKAP1 modulates the mitochondrial fission mediated by DRP1 phosphorylation.

### CaN mediates the phosphorylation of DRP1 promoted by Oip5-as1

**Fig. 6:**
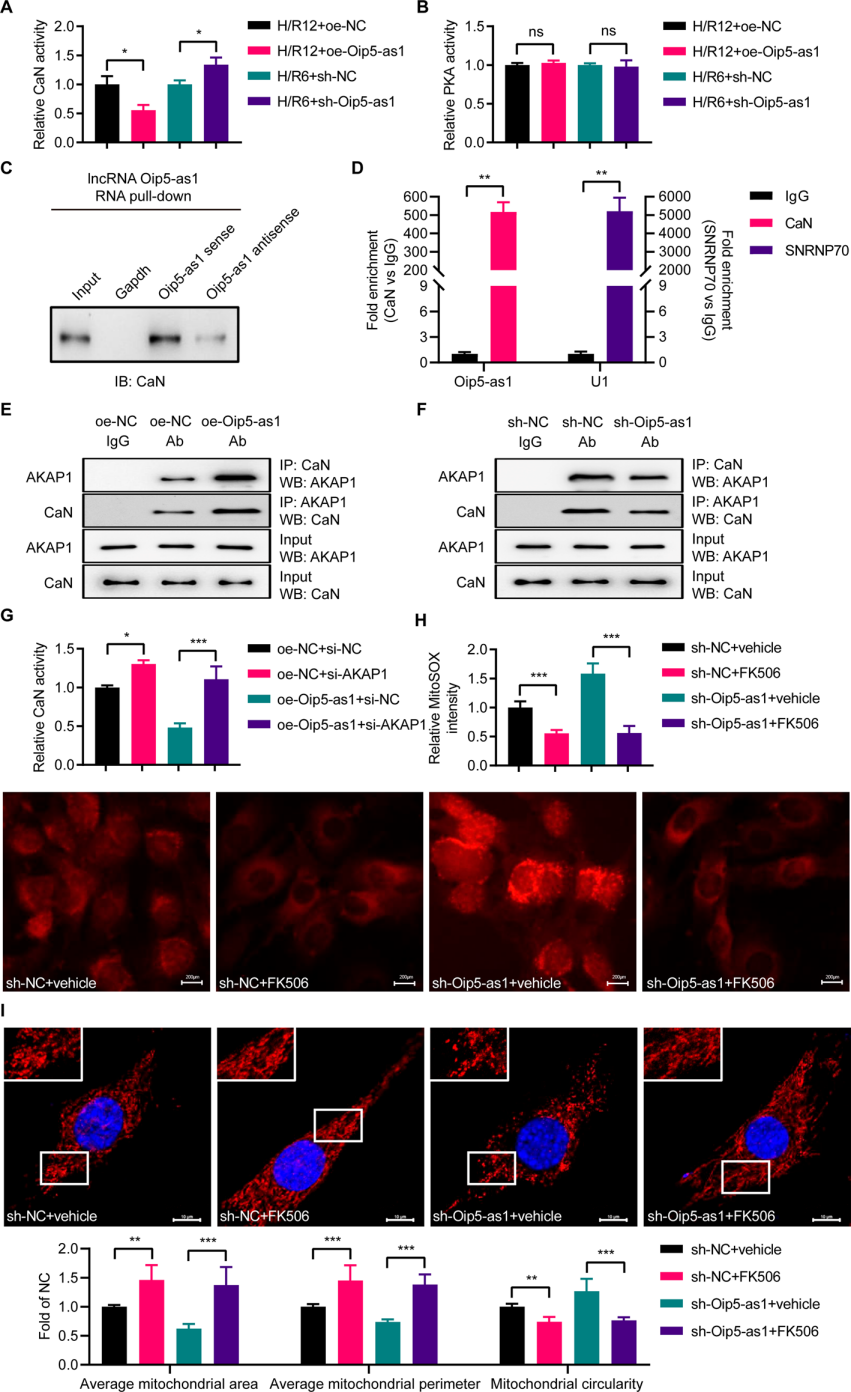
CaN mediates Oip5-as1-promoted DRP1 phosphorylation. **A** CaN activity is measured using the commercial assay kit in Oip5-as1-overexpressing or knockdown HL-1 cells during hypoxia/reoxygenation (H/R). **B** PKA activity is determined utilizing the commercial kit in Oip5-as1-overexpressing or knockdown HL-1 cells during H/R. **C** RNA pulldown assays are performed using biotin-labeled sense, antisense Oip5-as1 or Gapdh. Proteins from HL-1 cell extracts are immunoblotted with the CaN antibody. **D** RNA immunoprecipitation assays are conducted using the CaN antibody, followed by RT-qPCR to enrich Oip5-as1. Anti-SNRNP70 serves as a positive control. **E** Co-immunoprecipitation (co-IP) analysis probes interactions between AKAP1 and CaN in Oip5-as1-overexpressing HL-1 cells. **F** Co-IP analysis examines AKAP1 and CaN interactions in Oip5-as1-knockdown HL-1 cells. **G** Measurement of CaN activity in HL-1 cells during H/R is conducted. The results show that si-AKAP1 reverses the decrease in CaN activity induced by Oip5-as1 overexpression. **H** MitoSOX staining demonstrates that treatment with FK506 (a CaN inhibitor) reduces mitochondrial ROS levels in Oip5-as1-knockdown HL-1 cells subjected to H/R induction. Scale bar, 200 μm. **I** Analysis of mitochondrial morphology reveals that FK506 treatment increases the average mitochondrial area and perimeter, while decreasing circularity in Oip5-as1-knockdown HL-1 cells undergoing H/R induction. The mitochondria are visualized using an antibody specific to Tom-20, while the cell nuclei are stained with DAPI. The upper panels show enlarged views of the regions indicated by the boxes in the lower panels. Scale bar, 10 μm. For the Oip5-as1 overexpression experiment, an H/R12 protocol, comprising 6 h of hypoxia followed by 12 h of reoxygenation, was implemented. Conversely, in the Oip5-as1 knockdown experiment, an H/R6 protocol, consisting of 6 h of hypoxia followed by 6 h of reoxygenation, was adopted. Data are presented as mean ± standard deviation, *n* = 3. *P < 0.05, **P < 0.01, and ***P < 0.001. *CaN* calcineurin, *PKA* protein kinase A, *AKAP1* a kinase anchor protein 1, *Tom-20* translocase of outer mitochondria 20, *DAPI* 4', 6-diamidino-2-phenylindole, *ROS* reactive oxygen species, *RT-qPCR* real-time quantitative polymerase chain reaction, *si-AKAP1* small interfering RNAs targeting AKAP1, *si-NC* negative control small interfering RNAs, *oe-Oip5-as1* the overexpressed lentivirus for Oip5-as1, *oe-NC* the overexpressed lentivirus for negative control, *sh-Oip5-as1* the short hairpin RNA (shRNA)-mediated lentivirus targeting Oip5-as1, *sh-NC* the negative control shRNA lentivirus

AKAP1 has been demonstrated to regulate the levels of p-DRP1^Ser637^ by interacting with and modulating the activities of PKA and CaN. To delineate the signaling pathways implicated in Oip5-as1’s function, we separately assessed the activities of PKA and CaN in HL-1 cells with Oip5-as1 expression either upregulated or silenced, utilizing targeted assay kits. The assay results showed that CaN activity was reduced in the oe-Oip5-as1 group relative to the oe-NC group, whereas it was elevated in the sh-Oip5-as1 group compared to the sh-NC group (Fig. 6A). Notably, PKA activity did not exhibit significant variation when comparing both the overexpression groups (oe-Oip5-as1 versus oe-NC) and the knockdown groups (sh-Oip5-as1 versus sh-NC) (Fig. 6B).

Activated CaN has been shown to exacerbate MI/R injury by promoting the dephosphorylation of DRP1 at the Ser637 site. In this context, our study aimed to determine if Oip5-as1 could attenuate CaN activity by influencing its interaction with AKAP1. RNA pull-down assays showed that Oip5-as1, but not antisense Oip5-as1 or Gapdh, could bind to CaN (Fig. 6C). Correspondingly, RIP assays demonstrated specific immunoprecipitation of Oip5-as1 with CaN-specific antibodies, as opposed to control IgG antibodies (Fig. 6D).

Further co-IP assays revealed a differential interaction between CaN and AKAP1 depending on Oip5-as1 expression levels: overexpression of Oip5-as1 led to an increased co-precipitation of both proteins (Fig. 6E), whereas Oip5-as1 knockdown resulted in their reduced interaction (Fig. 6F). CaN activity assays indicated that si-AKAP1 enhanced CaN activity in HL-1 cells following H/R12 exposure, and mitigated the suppressive impact of Oip5-as1 overexpression on CaN activity (Fig. 6G).

We subsequently examine whether Oip5-as1 regulates mitochondrial fission in a CaN-dependent manner. In rescue experiments, FK506 intervention led to a significant reduction in mtROS levels in HL-1 cells subjected to both H/R6 injury and sh-Oip5-as1 knockdown (Fig. 6H). Furthermore, treatment with FK506 increased the average mitochondrial area and perimeter, decreased circularity, and counteracted the sh-Oip5-as1-induced changes in mitochondrial morphology (Fig. 6I). Taken together, the interaction among Oip5-as1, AKAP1, and CaN is crucial for regulating DRP1 phosphorylation and the process of mitochondrial fission.

### Knockout of Oip5-as1 exacerbates MI/R injury in mouse models

**Fig. 7:**
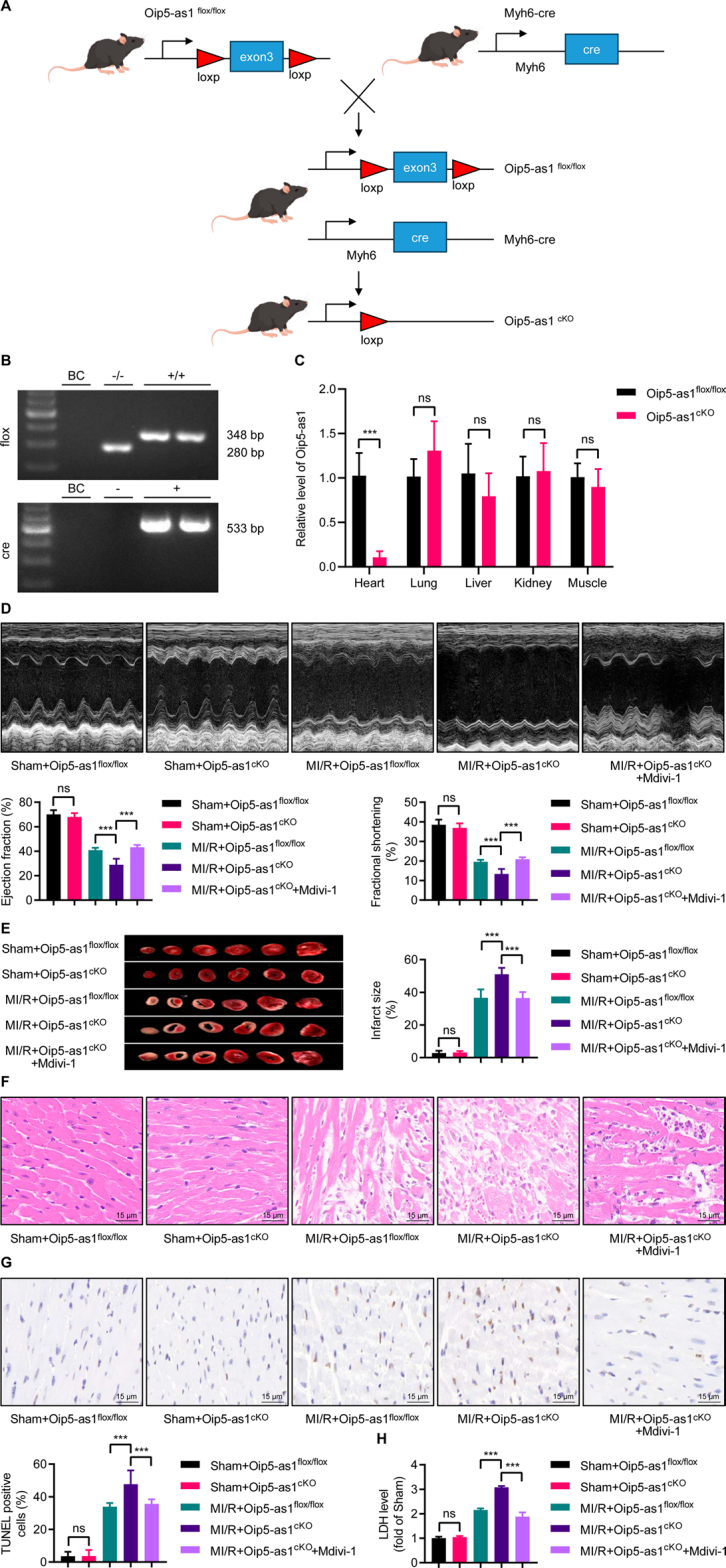
Oip5-as1 knockout exacerbates MI/R injury in mice. **A** A schematic outlines the strategy to generate cardiomyocyte-specific Oip5-as1 knockout (Oip5-as1^cKO^) mice. **B** PCR analysis of tail genomic DNA reveals the Oip5-as1 floxed allele (Oip5-as1^flox/flox^) and Cre recombinase gene. ddH_2_O as blank control (BC). **C** RT-qPCR confirms efficient Oip5-as1 knockout in the heart tissue of Oip5-as1^cKO^ mice, with no change in expression in other tissues like the lung, liver, kidney, and skeletal muscle. **D** Echocardiographic assessments, including images and measurements of left ventricular ejection fraction and fractional shortening, are performed in mice after MI/R injury. **E** TTC staining of heart sections is used to visualize and measure infarct size in mice following MI/R. **F** H&E staining is conducted on left ventricular sections from hearts injured by MI/R. Scale bar, 15 μm. **G** TUNEL staining of heart sections provides visualization and quantification of TUNEL-positive cells in left ventricular sections of hearts affected by MI/R. Scale bar, 15 μm. **H** Serum lactate dehydrogenase (LDH) levels are quantified in mice after MI/R injury. Data are presented as mean ± standard deviation, *n* = 6. ***P < 0.001. *ns* nonstatistically significant, *MI/R* myocardial ischemia/reperfusion, *RT-qPCR* real-time quantitative polymerase chain reaction, *Mdivi-1* mitochondrial division inhibitor-1, *TTC* 2,3,5-triphenyltetrazolium chloride, *H&E* hematoxylin and eosin staining, *TUNEL* terminal deoxynucleotidyl transferase dUTP nick end labeling

To explore the potential adverse effects of reduced Oip5-as1 expression on cardiac function in vivo, we generated cardiomyocyte-specific Oip5-as1 knockout (Oip5-as1^flox/flox^, Cre^Myh6^; Oip5-as1^cKO^) mice using CRISPR/Cas9 genome editing (Fig. 7A). Figure 7B presents gel electrophoresis results, where the PCR product from flox-specific primers matches the anticipated 348 bp size for the knocked-out allele, in contrast to the 280 bp size of the wild-type allele. Additionally, the Cre-specific PCR product is in line with the 533 bp size expected for the Cre transgene fragment (Fig. 7B). In Oip5-as1^cKO^ mice, myocardial Oip5-as1 expression was depleted, with levels in the liver, lung, spleen, and skeletal muscle remaining unchanged according to RT-qPCR analysis (Fig. 7C).

In the MI/R model, Oip5-as1^cKO^ mice demonstrated significant cardiac dysfunction, with reduced LVEF and LVFS, relative to Oip5-as1^flox/flox^ controls (Fig. 7D). This cardiac impairment correlated with increased infarct size in Oip5-as1^cKO^ mice, as indicated by TTC staining (Fig. 7E). H&E staining revealed that the myocardial damage in Oip5-as1^cKO^ mice was more severe, with heightened inflammatory infiltration, cellular disarray, and fibrosis (Fig. 7F). Moreover, TUNEL assays showed higher rates of apoptosis in Oip5-as1-deficient myocardium post-MI/R (Fig. 7G). Serum LDH levels were also elevated in Oip5-as1^cKO^ mice, indicating more severe cellular damage (Fig. 7H). These findings collectively suggest a protective role for Oip5-as1 in the heart following MI/R injury.

Treatment with Mdivi-1 in Oip5-as1^cKO^ mice following MI/R significantly improved cardiac function and decreased infarct size (Fig. 7D, E). This was accompanied by reduced myocardial damage and inflammation, as evidenced by H&E staining (Fig. 7F). The TUNEL assay also showed a significant reduction in apoptosis rates (Fig. 7G). Furthermore, decreased serum LDH activity in the treated mice suggested ameliorated cellular damage (Fig. 7H). The data indicate that inhibiting DRP1 effectively reverses cardiac injury following MI/R in Oip5-as1^cKO^ mice.

### Oip5-as1^cKO^ mice exhibit excessive mitochondrial fission mediated by DRP1 activation

**Fig. 8:**
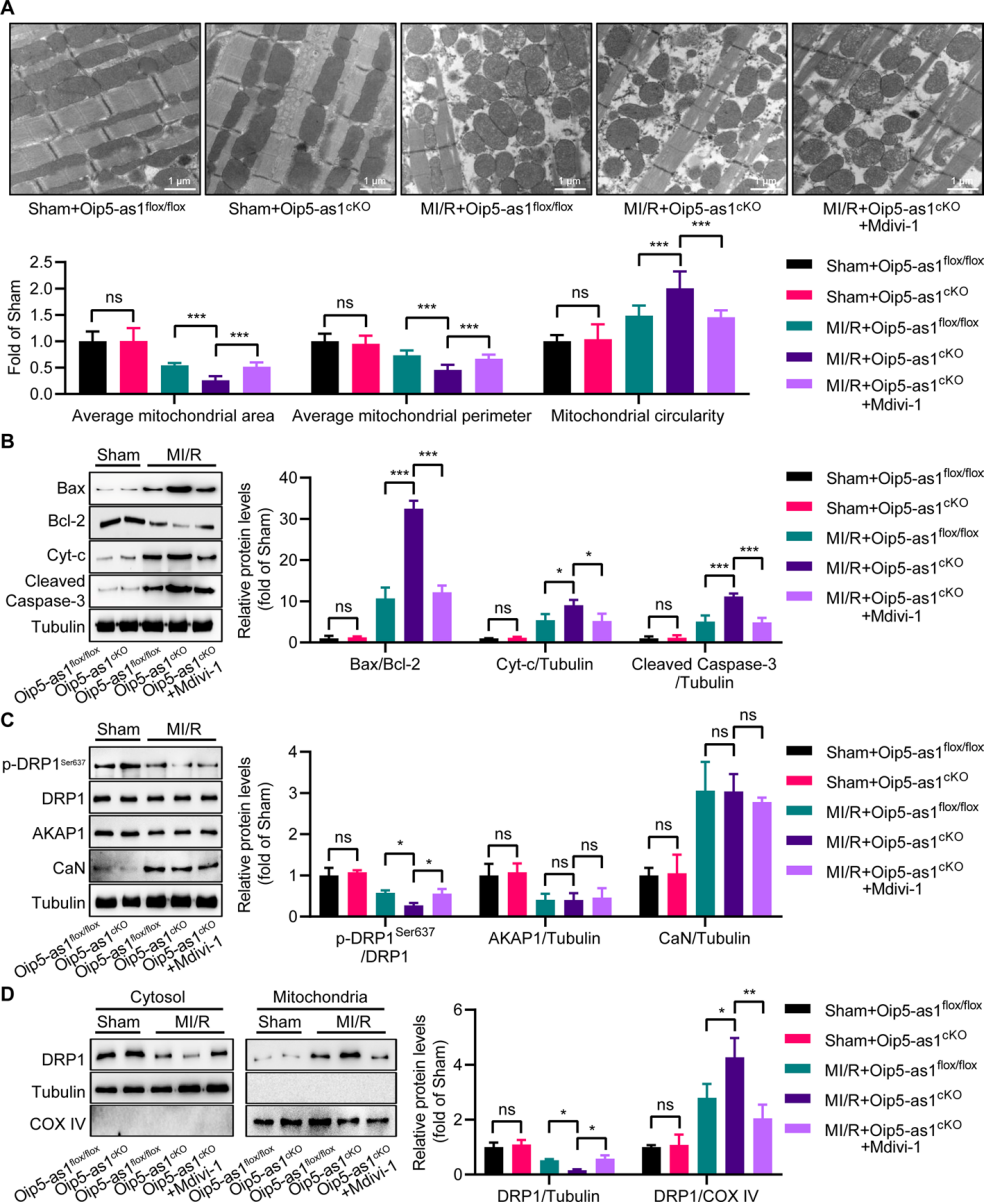
Excessive mitochondrial fission mediated by DRP1 in Oip5-as1 knockout mice during MI/R process. **A** Representative TEM images display mitochondrial morphology in the myocardium following MI/R, with quantitative analysis including measurements of average mitochondrial area, perimeter, and circularity. **B** Western blot and quantitative analyses reveal the expression levels of mitochondrial-associated apoptotic proteins in the myocardium following MI/R, including Bax/Bcl-2, Cyt-c, and Cleaved-Caspase-3. **C** Western blot and quantitative analyses determine changes in protein levels of p-DRP1^Ser637^, AKAP1, and CaN in the myocardium of mice post-MI/R injury. **D** Western blot and quantitative analyses of DRP1 localization in the myocardium post MI/R reveal its distribution between cytosol and mitochondria. COX IV protein serves as a mitochondrial marker to identify mitochondrial fractions, while Tubulin protein is used as a cytoplasmic marker for cytoplasmic fractions. Data are presented as mean ± standard deviation, *n* = 3. *P < 0.05, **P < 0.01, and ***P < 0.001. *ns* nonstatistically significant, *MI/R* myocardial ischemia/reperfusion, *Oip5-as1*^*cKO*^ cardiomyocyte-specific Oip5-as1 knockout, *Oip5-as1*^*flox/flox*^ Oip5-as1 floxed allele, *Mdivi-1* mitochondrial division inhibitor-1 (an inhibitor of DRP1), *TEM* transmission electron microscopy, *Bax* Bcl-2 associated X protein, *Bcl-2* B-cell lymphoma 2, *Cyt-c* cytochrome c, *AKAP1* a kinase anchor protein 1, *CaN* calcineurin, *DRP1* dynamin-related protein 1, *COX IV* cytochrome c oxidase subunit IV

TEM was utilized to assess myocardial mitochondrial morphology. In the MI/R model, Oip5-as1^cKO^ mice demonstrated a significant decrease in both average mitochondrial area and perimeter when compared to Oip5-as1^flox/flox^ controls, alongside an increased circularity (Fig. 8A). Notably, the administration of Mdivi-1 mitigated the enhanced mitochondrial fission observed in the Oip5-as1^cKO^ group (Fig. 8A). Western blot analysis revealed that Oip5-as1^cKO^ mice, within the MI/R model, had significantly higher expression levels of mitochondrial-associated apoptotic proteins, including Bax/Bcl-2, Cyt-c, and Cleaved-Caspase-3, than the Oip5-as1^flox/flox^ group (Fig. 8B). Administration of Mdivi-1, however, countered this upregulation in the Oip5-as1^cKO^ mice (Fig. 8B).

Mechanistic analysis showed that Oip5-as1^cKO^ reduced the expression level of p-DRP1^Ser637^, while it did not affect the expression of AKAP1 and CaN (Fig. 8C). Following the administration of Mdivi-1 treatment to Oip5-as1^cKO^ mice, an increase in the expression level of p-DRP1^Ser637^ was observed, whereas the expression of AKAP1 and CaN remained unaffected (Fig. 8C). Western blot assays indicated that Oip5-as1^cKO^ mice exhibited a significant increase in mitochondrial DRP1 expression following MI/R injury, compared to Oip5-as1^flox/flox^ mice (Fig. 8D). Additionally, treatment with Mdivi-1 significantly reduced mitochondrial DRP1 expression in Oip5-as1^cKO^ mice (Fig. 8D). The findings suggest that Oip5-as1 knockout exacerbates mitochondrial fission induced by MI/R through the suppression of p-DRP1^Ser637^ levels.

### Overexpression of Oip5-as1 attenuates MI/R injury in mouse models

**Fig. 9:**
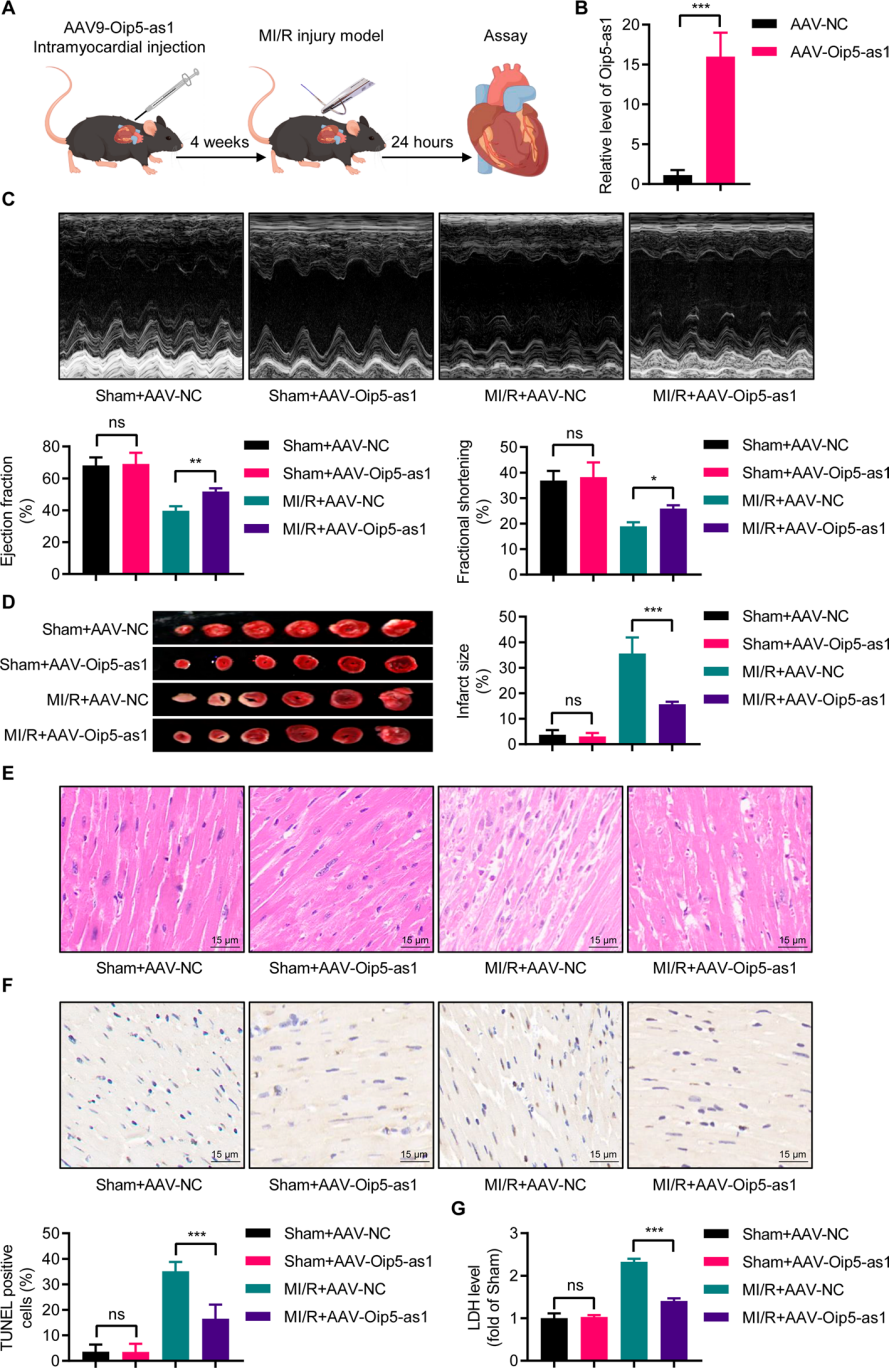
Oip5-as1 overexpression alleviates MI/R injury in mice. **A** Schematic plot illustrating the experimental design, where Oip5-as1 overexpression is achieved through AAV9 delivery in mice. **B** RT-qPCR analysis demonstrating significant elevation in Oip5-as1 gene expression in the myocardium of the AAV9-Oip5-as1 group compared to the AAV-NC. **C** Representative echocardiographic images and statistical data showing improved left ventricular ejection fraction and fractional shortening in Oip5-as1 overexpression mice post MI/R injury. **D** Representative TTC staining images and quantified infarct size data in mice post MI/R injury, indicating the protective effects of Oip5-as1 overexpression against MI/R damage. **E** H&E staining of left ventricular sections from Oip5-as1 overexpression mice post MI/R injury, showing histological evidence of tissue preservation. Scale bar, 15 μm. **F** TUNEL assay images and quantitative analysis showing reduced apoptotic cardiomyocytes in heart sections of Oip5-as1 overexpression mice. Scale bar, 15 μm. **G** Detection of serum cardiac injury marker LDH in Oip5-as1 overexpression mice post MI/R injury, serving as biochemical evidence of reduced cardiac damage. Data are presented as mean ± standard deviation, *n* = 6. *P < 0.05, **P < 0.01, and ***P < 0.001, *ns* nonstatistically significant, *MI/R* myocardial ischemia/reperfusion, *AAV9* adeno-associated virus serotype 9, *AAV-Oip5-as1* recombinant AAV9 vectors for Oip5-as1 overexpression, *AAV-NC* recombinant AAV9 vectors for a negative control, *RT-qPCR* real-time quantitative polymerase chain reaction, *TTC* 2,3,5-triphenyltetrazolium chloride, *H&E* hematoxylin and eosin staining, *TUNEL* terminal deoxynucleotidyl transferase dUTP nick end labeling, *LDH* lactate dehydrogenase

To assess the impact of Oip5-as1 overexpression on MI/R injury, we employed AAV9-mediated gene delivery to upregulate Oip5-as1 in the mouse model (Fig. 9A). RT-qPCR analysis revealed a significant 15.9-fold increase in Oip5-as1 expression levels in the AAV-Oip5-as1 group compared to the AAV-NC group, as shown in Fig. 9B. Echocardiographic analysis showed that AAV-Oip5-as1 treatment improved cardiac function compared to AAV-NC, as evidenced by increased LVEF and LVFS (Fig. 9C). TTC staining confirmed a reduced infarct size in the AAV-Oip5-as1 group (Fig. 9D). Moreover, H&E and TUNEL assays revealed less myocardial damage (Fig. 9E) and apoptosis (Fig. 9F), respectively, and LDH assays indicated lower cellular damage in the AAV-Oip5-as1 group than in the AAV-NC group (Fig. 9G). These results demonstrate that targeted overexpression of Oip5-as1 mitigates the extent of cardiac injury induced by MI/R.

### Oip5-as1 upregulation inhibits mitochondrial fission through DRP1 phosphorylation in mice

**Fig. 10:**
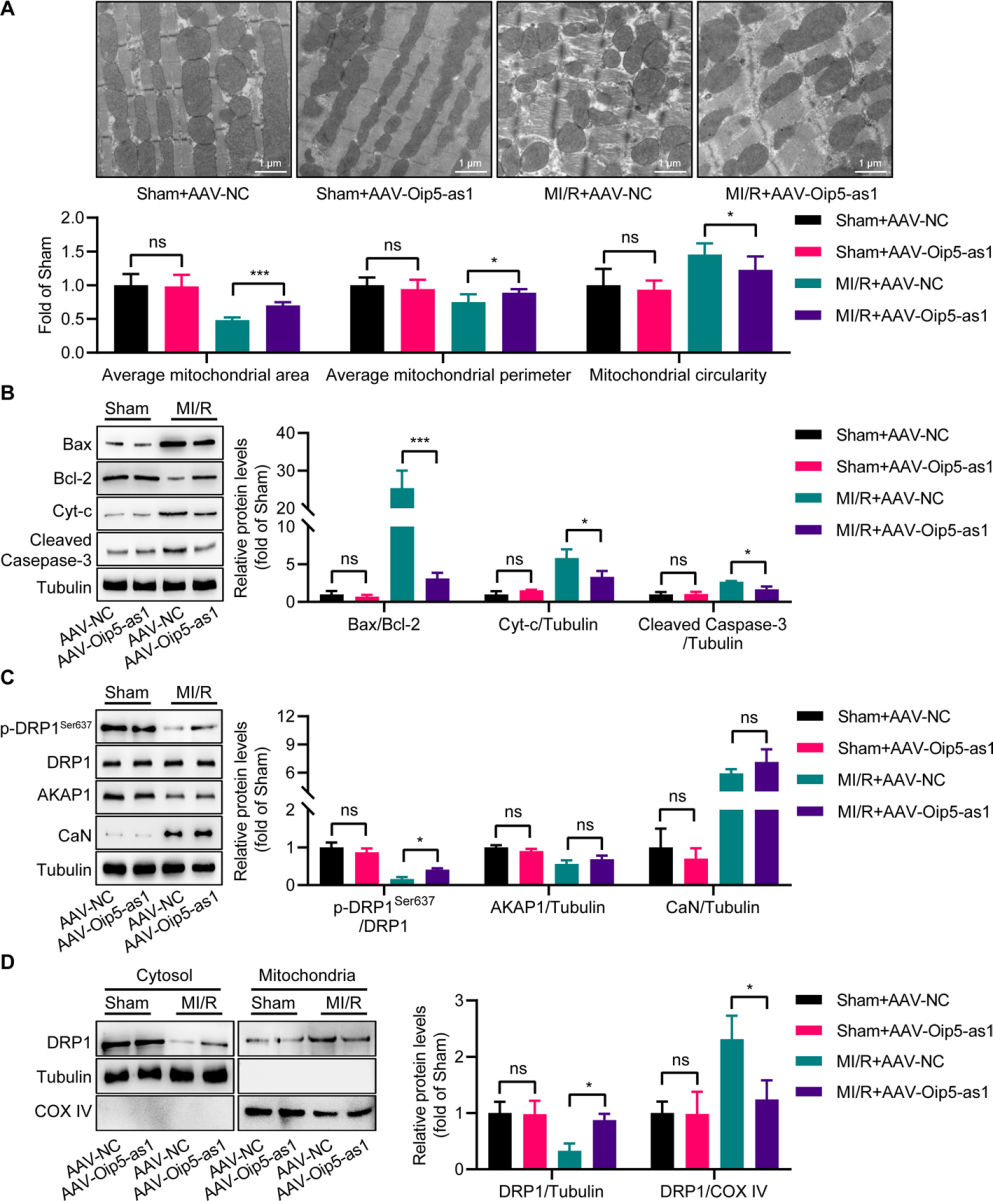
Oip5-as1 overexpression inhibits mitochondrial fission in mouse myocardium after MI/R by promoting DRP1 phosphorylation. **A** Representative transmission electron microscopy (TEM) images exhibit mitochondrial morphology in myocardium of mice with Oip5-as1 overexpression post MI/R. Quantitative analyses of average mitochondrial area, perimeter, and circularity are shown. **B** Western blot and quantitative analyses of mitochondrial apoptosis-related proteins in the myocardium of mice with Oip5-as1 overexpression after MI/R, including Bax/Bcl-2, Cyt-c, and Cleaved-Caspase-3. **C** Western blot and quantitative analyses display the protein levels of p-DRP1^Ser637^, AKAP1, and CaN in Oip5-as1 overexpressing mice following MI/R injury. **D** Subcellular distributions of DRP1 in cytosolic and mitochondrial fractions of mice with Oip5-as1 overexpression after MI/R injury assessed by Western blot and quantitative analyses. COX IV protein serves as a mitochondrial marker for identifying mitochondrial fractions, while Tubulin protein is used as a cytoplasmic marker to distinguish cytoplasmic fractions. Data are presented as mean ± standard deviation, *n* = 3. *P < 0.05, ***P < 0.001. *ns* nonstatistically significant, *MI/R* myocardial ischemia/reperfusion, *AAV-Oip5-as1* recombinant AAV9 vectors for Oip5-as1 overexpression, *AAV-NC* recombinant AAV9 vectors for a negative control, *Bax* Bcl-2 associated X protein, *Bcl-2* B-cell lymphoma 2, *Cyt-c* cytochrome c, *AKAP1* a kinase anchor protein 1, *CaN* calcineurin, *DRP1* dynamin-related protein 1, *COX IV* cytochrome c oxidase subunit IV

In mice with MI/R injury, TEM revealed that AAV-Oip5-as1 treatment significantly increased the average mitochondrial area and perimeter, and decreased circularity, compared to AAV-NC (Fig. 10A). Western blot analysis showed that AAV-Oip5-as1 significantly reduced the expression levels of mitochondrial pro-apoptotic proteins (Bax/Bcl-2 ratio, Cyt-c, and Cleaved-Caspase-3) in these mice (Fig. 10B).

Mechanistic analysis revealed that under MI/R conditions, Oip5-as1 overexpression significantly increased p-DRP1^Ser637^ levels without affecting the abundance of AKAP1 and CaN, compared to the AAV-NC group (Fig. 10C). Western blot analysis demonstrated that AAV-Oip5-as1 treatment led to a significant reduction in mitochondrial DRP1 levels following MI/R injury compared to the AAV-NC group, as evidenced by Fig. 10D. These findings indicate that Oip5-as1 upregulation during MI/R injury can inhibit DRP1-mediated mitochondrial fission.

## Discussion

**Fig. 11:**
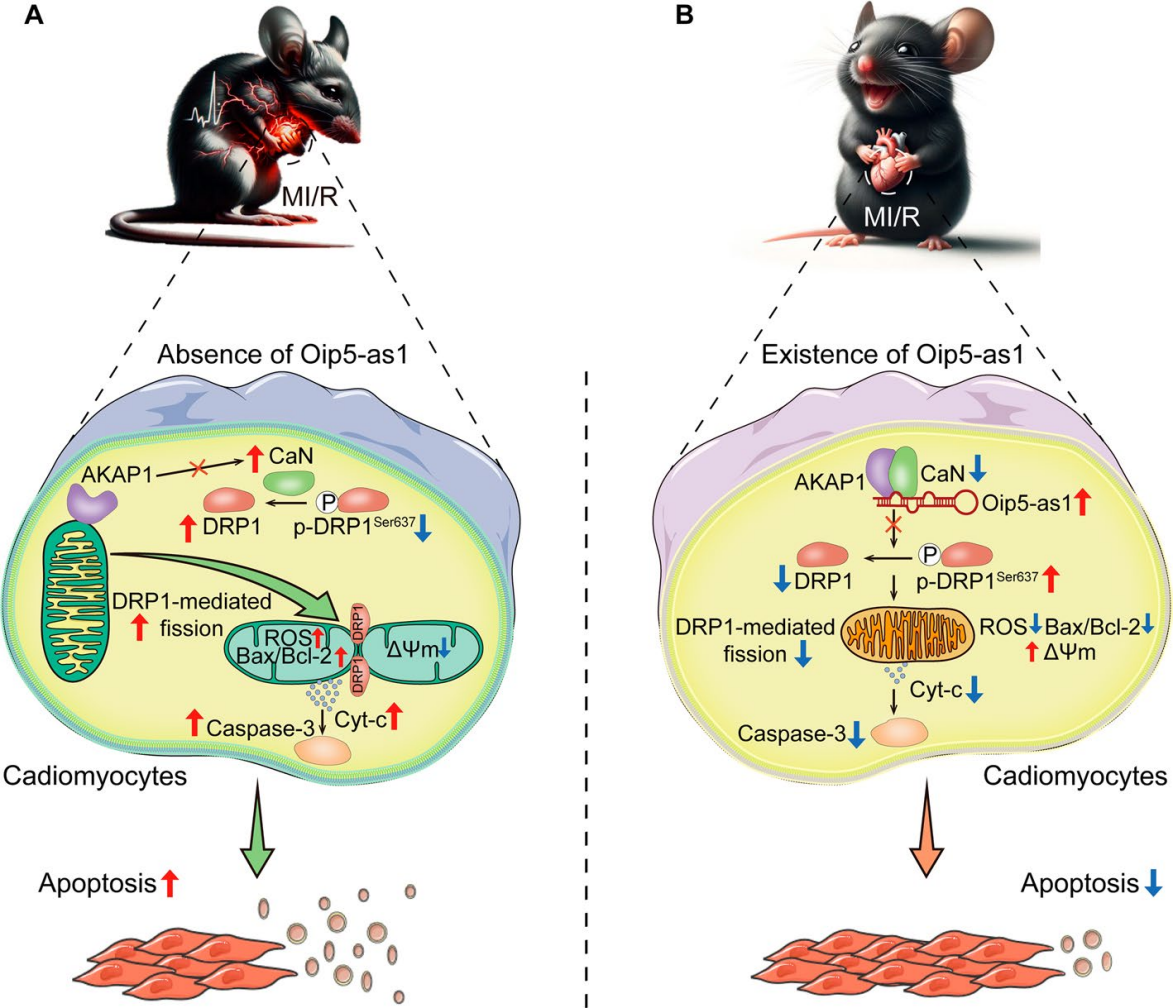
Proposed mechanisms underlying the protective effects of Oip5-as1 on MI/R injury. **A** During myocardial ischemia/reperfusion (MI/R), the expression of Oip5-as1 is decreased. This affects AKAP1’s inhibition of calcineurin (CaN), leading to increased CaN-mediated dephosphorylation of dynamin-related protein 1 (DRP1) at serine 637 (p-DRP1^Ser637^). As a result, more translocation of DRP1 from cytoplasm to the outer mitochondrial membrane is promoted. This induces excessive mitochondrial fission and dysfunction, evidenced by increased mitochondrial reactive oxygen species (ROS), increased Bcl-2 associated X protein (Bax)/ B-cell lymphoma 2 (Bcl-2) ratio and decreased mitochondrial membrane potential (Δ*Ψ*m). Consequently, the release of the pro-apoptotic factor cytochrome C (Cyt-c) is provoked, as well as the activation of the apoptosis executioner Caspase-3. Ultimately, apoptotic injury of cardiomyocytes is caused. **B** When Oip5-as1 is overexpressed, it recruits CaN to AKAP1, thereby inhibiting CaN activation. This results in increased levels of p-DRP1^Ser637^, preventing DRP1 translocation to the mitochondria and mitochondrial morphological and functional impairments mediated by DRP1. Consequently, the downstream release of Cyt-c and activation of Caspase-3 are decreased. Ultimately, the incidence of cardiomyocyte apoptosis is reduced

This study revealed that Oip5-as1 expression is downregulated during MI/R injury. Overexpression of Oip5-as1 significantly attenuates myocardial apoptosis resulting from excessive mitochondrial fission, whereas Oip5-as1 knockdown exacerbates this injury. Further mechanistic analyses have demonstrated that the protective effect of Oip5-as1 is mediated by its role as a molecular scaffold for AKAP1 and CaN. This interaction enhances p-DRP1^Ser637^ levels and prevents DRP1 translocation to the mitochondria (Fig. 11).

lncRNAs are key regulators in the pathogenesis of MI/R injury, influencing processes such as cell death, fibrosis, and inflammation [18]. Emerging research has started to unveil a potential link between lncRNAs and mitochondrial dynamics. For example, lncRNA CARL can mitigate MI/R injury by targeting the mitochondrial fission mediator PHB2, thereby reducing mitochondrial fission [32]. Conversely, lncRNA PVT1 is implicated in exacerbating MI/R injury by promoting mitochondrial fragmentation [33]. Collectively, these findings highlight the potential of lncRNAs as therapeutic targets for modulating mitochondrial fission and fusion, which are essential for maintaining mitochondrial integrity and function in the context of MI/R injury. Oip5-as1 is involved in a multitude of functions in both physiological and pathological contexts, including neurological disorders, cancers, and inflammatory conditions [26]. Recent advancements using CRISPR technology have led to the development of a global Oip5-as1 knockout mouse model, with findings indicating that female KO mice suffer from aggravated heart failure following cardiac pressure overload, as induced by transverse aortic constriction [25]. Comparative RNA-sequencing analysis of wild-type and knockout hearts suggests that Oip5-as1 may play a role in modulating pathways critical to mitochondrial function [33]. Concurrently, our research group has identified a cardioprotective role for Oip5-as1 in MI/R injury [22]. However, the specific molecular mechanisms by which Oip5-as1 confers protection against MI/R injury have not yet been elucidated. In this study, we used loss- and gain-of-function approaches in both in vitro and in vivo experiments to demonstrate that Oip5-as1 inhibits myocardial apoptosis induced by excessive mitochondrial fission. Subsequent rescue experiments identified that the cardioprotective role of Oip5-as1 is dependent on the level of p-DRP1^Ser637^.

Gene knockout studies have revealed that the phosphorylation state of DRP1 at Ser637 is crucial for its activity [27, 28, 34]: dephosphorylation leads to activation, whereas phosphorylation results in inhibition. Upon activation, DRP1 translocates to the outer mitochondrial membrane, where it binds to receptor proteins including Fis1, Mff, MIEF1, MiD49, and MiD51. DRP1 then oligomerizes into a ring structure that constricts around the mitochondrion, ultimately dividing it into two separate organelles. DRP1-mediated fission is advantageous for cellular function when maintained at physiological levels. However, excessive mitochondrial fission can escalate mtROS production, trigger the release of pro-apoptotic factors, and compromise mitochondrial function, contributing to increased myocardial apoptosis and cardiac dysfunction during MI/R events [4, 6]. Pharmacological research has shown that modulating p-DRP1^Ser637^ levels with specific agents can affect mitochondrial fission and function during the MI/R process [10, 35, 36]. Our study confirms that p-DRP1^Ser637^ levels influence the balance of mitochondrial fission. Notably, we have discovered that Oip5-as1 is a crucial upstream regulator of p-DRP1^Ser637^, an insight of importance given the limited number of studies on lncRNAs that regulate p-DRP1^Ser637^ during MI/R injury.

To further elucidate the mechanism by which Oip5-as1 promotes DRP1 phosphorylation, we employed molecular interaction assays such as RNA pull-down, RIP, and co-IP. The assays indicated that Oip5-as1 can directly interact with AKAP1 and CaN, thereby significantly increasing p-DRP1^Ser637^ levels. These findings offer new insights into the phosphorylation dynamics of DRP1 in MI/R injury. AKAP1 is a pivotal regulator of mitochondrial function, coordinating the assembly of signaling proteins and RNA on the outer mitochondrial membrane [37]. In cardiac cells, AKAP1-mediated recruitment of PKA helps to prevent mitochondrial fragmentation and dysfunction, thereby contributing to cardioprotection [38]. Furthermore, AKAP1’s interaction with CaN diminishes the cytosolic availability of active CaN, which in turn helps to reduce mitochondrial fission and dysfunction [39, 40]. Previous studies have indicated that CaN activates DRP1 by dephosphorylation [27], whereas PKA inhibits DRP1 through phosphorylation [34]. The fine-tuned regulatory mechanisms that align the effects of CaN and PKA on DRP1 with specific cellular activities are, however, not well understood [41]. Our study sheds light on this by demonstrating that Oip5-as1 can selectively regulate CaN-mediated dephosphorylation of DRP1 without interfering with PKA’s phosphorylation activity.

CaN is a conserved protein phosphatase that specifically targets phosphoserine and phosphothreonine residues [42]. CaN is typically inactive in the non-stimulated heart, a state maintained by two primary mechanisms [42]. The first involves an auto-inhibitory domain that blocks CaN’s active site, which is displaced when Ca^2+^/calmodulin binds to the enzyme in response to intracellular signals. The second mechanism involves inhibitory proteins, such as AKAP1 and RCAN1, which bind to and suppress CaN’s activity. Once activated, CaN dephosphorylates DRP1 at Ser637, which facilitates DRP1’s translocation to the mitochondria and promotes mitochondrial fission [27]. Activation of CaN is known to exacerbate ischemia/reperfusion-induced damage in the heart and brain [43]. Conversely, treatment with the CaN inhibitor FK506 prevents DRP1 dephosphorylation at Ser637, preserving cardiac function [36]. In the rescue experiments conducted in this study, we established that Oip5-as1’s regulatory role on DRP1-mediated mitochondrial fission depends on the activity of CaN. Moreover, co-IP experiments have demonstrated that Oip5-as1 enhances the interaction between AKAP1 and CaN. Consequently, Oip5-as1 acts as a molecular scaffold, sequestering CaN to specifically attenuate its dephosphorylation effect on DRP1, a mechanism that aligns with the observed decrease in mitochondrial fission. Overall, this study is the first to reveal that Oip5-as1 directly interacts with the AKAP1-CaN complex, thereby enhancing DRP1 phosphorylation and inhibiting the excessive mitochondrial fission induced by MI/R.

lncRNAs exhibit versatility in interacting with proteins, a trait attributed to their complex and adaptable structures [19]. These molecules can adopt various conformations, such as hairpins and loop formations, creating multiple binding sites for proteins. The modular domains and dynamic structures of lncRNAs allow for both simultaneous and sequential binding to diverse interaction partners. For instance, conformational changes in response to cellular conditions facilitate the formation of intricate lncRNA-protein assemblies and enable precise base pairing between lncRNAs and protein RNA-binding domains [44, 45]. Such structural adaptability empowers lncRNAs to function as molecular scaffolds that mediate protein interactions and phosphorylation events affecting downstream pathways [44, 45]. Studies have reported that Oip5-as1 binds to various proteins, thereby modulating the expression of downstream mRNAs. Kim et al. [46] showed that Oip5-as1’s interaction with the HuR protein, an ELAV family member, stabilizes and regulates the translation of specific mRNAs. Additionally, Wanowska et al. [47] found that Oip5-as1 binds directly to SMARCA4, a key component of the SWI/SNF chromatin remodeling complex, influencing chromatin configuration and the transcription of certain mRNAs. Together, these studies underscore Oip5-as1’s critical role in the complex network of protein interactions that maintain cellular homeostasis and regulate gene expression. In this study, we report that Oip5-as1 can simultaneously bind to AKAP1 and CaN, uncovering a previously undefined role of Oip5-as1 in post-translational regulation. This dual interaction underscores Oip5-as1’s role as a molecular scaffold and suggests its potential to coordinate the functions of these proteins. These findings open avenues for further research into Oip5-as1’s involvement in cellular regulatory networks and its contribution to diseases where AKAP1 and CaN are key.

Recent research utilizing advanced methods like gene knockout, transgenic models, and AAV9 mediated gene editing has revealed the therapeutic potential of lncRNAs in MI/R injury. Studies by Zhang et al. [48] and Gao et al. [21] have been pivotal in showing how lncRNAs, such as CIRBIL and CPhar, modulate cardiac function and injury response through techniques like CRISPR/Cas9-mediated knockout and AAV9-mediated gene delivery. Building on these insights, our research further elucidates the significant role of Oip5-as1. In our mouse models, we found that alterations in Oip5-as1 expression significantly impacted mitochondrial fission associated with MI/R injury, in line with its regulatory functions at the cellular level. Specifically, Oip5-as1 knockout in mice led to increased infarct size and impaired cardiac function, while its overexpression improved cardiac outcomes. These changes, driven by DRP1 phosphorylation, establish Oip5-as1 as a key player in MI/R injury pathways. Our findings thus support the potential of Oip5-as1 as a promising therapeutic target in MI/R injury, offering novel approaches to alleviate its pathological effects.

Our study has several limitations. Firstly, our study employs cell and mouse models to explore the functions of Oip5-as1. These models are crucial for deciphering basic biological processes, yet the extent to which our findings can be applied to human physiology remains uncertain. This is largely attributed to potential variances in cardiac physiology and molecular responses between mice and humans. However, it should be noted that the mouse model is frequently regarded as a reliable proxy in this area of research, offering significant insights into human physiology, albeit not conclusively. Second, our focus on Oip5-as1, specifically its role in mitochondrial fission and cardiac function during MI/R injury, may not fully capture the condition’s multifaceted nature influenced by genetic, molecular, and environmental factors. Nevertheless, our research provides crucial preliminary insights into Oip5-as1’s role, forming a foundation for broader future investigations. Third, the detailed investigation of Oip5-as1’s interactions with proteins such as AKAP1 and CaN requires advanced structural biology techniques like X-ray crystallography, nuclear magnetic resonance spectroscopy, and cryogenic electron microscopy. While our study does not utilize these techniques, the results we have obtained offer a foundational understanding that future studies can build upon using these advanced methods. Lastly, in-depth in vivo studies are important for a more comprehensive understanding of Oip5-as1’s function in MI/R injury. Our current findings, although limited in this regard, provide significant insights that will guide and inform future in vivo investigations.

## Conclusions

This study presents a detailed analysis of lncRNA Oip5-as1, highlighting its pivotal role in mitigating MI/R injury. Oip5-as1 functions as a molecular scaffold between AKAP1 and CaN, inhibiting CaN-mediated DRP1 dephosphorylation at Ser637. This curtails DRP1’s translocation to the mitochondria and the associated excessive mitochondrial fission and dysfunction, thereby reducing cardiomyocyte apoptosis and preserving cardiac function after MI/R. These findings not only deepen our understanding of the molecular mechanisms involved in MI/R injury but also position Oip5-as1 as a promising therapeutic target for ischemia–reperfusion-related conditions. Future research could further explore the therapeutic potential of Oip5-as1 in treating human cardiac injuries.

### Supplementary Information

**Additional file 1: Table S1** Primer sequences used in this study. **Table S2** Information of antibodies used in this study.

**Additional file 2: Fig. S1** Effects of Oip5-as1 on mitochondrial dynamics in HL-1 cells under normal culture conditions. **A** Mitochondrial morphology analysis of HL-1 cells transfected with oe-Oip5-as1 or sh-Oip5-as1. Data from representative images and quantification of Tom-20-stained HL-1 cells show no significant changes in mitochondrial morphology (average mitochondrial area, perimeter, and circularity) upon oe-Oip5-as1 or sh-Oip5-as1 transfection compared to oe-NC or sh-NC controls. The cell nuclei are stained with DAPI. Scale bar, 10 μm. **B** Mitochondrial-derived ROS levels in HL-1 cells transfected with oe-Oip5-as1 or sh-Oip5-as1. MitoSOX staining shows no significant differences in fluorescence intensity between oe-Oip5-as1 or sh-Oip5-as1 transfected groups and oe-NC or sh-NC controls. Scale bar, 200 μm. **C** Mitochondrial membrane potential changes in HL-1 cells transfected with oe-Oip5-as1 or sh-Oip5-as1. JC-1 staining shows no significant alterations in the aggregate/monomer fluorescence intensity ratio of HL-1 cells with oe-Oip5-as1 or sh-Oip5-as1 transfection compared to oe-NC or sh-NC controls. Scale bar, 200 μm. Data are presented as mean ± standard deviation, *n* = 3. *ns* nonstatistically significant. *Tom-20* translocase of outer mitochondria 20. *DAPI* 4', 6-diamidino-2-phenylindole. *ROS* reactive oxygen species. *oe-Oip5-as1* the overexpressed lentivirus for Oip5-as1. *oe-NC* the overexpressed lentivirus for negative control. *sh-Oip5-as1* the short hairpin RNA (shRNA)-mediated lentivirus targeting Oip5-as1. *sh-NC* the negative control shRNA lentivirus

## Data Availability

The datasets supporting the conclusions of this article are included within the article and its additional files.

## Abbreviations

AAV9: Adeno-associated virus serotype 9
AAV-NC: Recombinant AAV9 vectors for a negative control
AAV-Oip5-as1: Recombinant AAV9 vectors for Oip5-as1 overexpression
Actb: Actin beta
AKAP1: A kinase anchor protein 1
Bax: Bcl-2 associated X protein
Bcl-2: B-cell lymphoma 2
CaN: Calcineurin
CCK-8: Cell Counting Kit-8
Co-IP: Co-immunoprecipitation
COX IV: Cytochrome c oxidase subunit IV
Cyt-c: Cytochrome c
DAPI: 4ʹ 6-Diamidino-2-phenylindole
DRP1: Dynamin-related protein 1
FISH: Fluorescence in situ hybridization
FITC: Fluorescein lsothiocyanate
H&E: Hematoxylin and eosin staining
H/R: Hypoxia/reoxygenation
LDH: Lactate dehydrogenase
lncRNAs: Long noncoding RNAs
Mdivi-1: Mitochondrial division inhibitor-1
MI/R: Myocardial ischemia/reperfusion
NC: Negative control
Neat1: Nuclear paraspeckle assembly transcript 1
ns: Nonstatistically significant
oe-NC: The overexpressed lentivirus for negative control
oe-Oip5-as1: The overexpressed lentivirus for Oip5-as1
Oip5-as1: Opa-interacting protein 5-antisense transcript 1
Oip5-as1^cKO^: Cardiomyocyte-specific Oip5-as1 knockout
Oip5-as1^flox/flox^: Oip5-as1 floxed allele
p-DRP1^Ser637^: DRP1 phosphorylation at the Ser637 position
PI: Propidium iodide
PKA: Protein kinase A
RIP: RNA immunoprecipitation
ROS: Reactive oxygen species
RT-qPCR: Real-time quantitative polymerase chain reaction
sh-NC: The negative control shRNA lentivirus
sh-Oip5-as1: The short hairpin RNA (shRNA)-mediated lentivirus targeting Oip5-as1
siRNAs: Small interfering RNAs
si-AKAP1: Small interfering RNAs targeting AKAP1
si-NC: Negative control small interfering RNAs
TEM: Transmission electron microscopy
Tom-20: Translocase of outer mitochondria 20
TTC: 2,3,5-Triphenyltetrazolium chloride
TUNEL: Terminal deoxynucleotidyl transferase dUTP nick end labeling
