# Supplementary material for: lncRNA Oip5-as1 inhibits excessive mitochondrial fission in myocardial ischemia/reperfusion injury by modulating DRP1 phosphorylation
Source: Cell Mol Biol Lett. 2024 May 14;29:72. doi: 10.1186/s11658-024-00588-4 (PMC11092055; doi:10.1186/s11658-024-00588-4)
Supplement: Supplementary file 1 — Additional file 1: Table S1 Primer sequences used in this study. Table S2 Information of antibodies used in this study. [file 11658_2024_588_MOESM1_ESM.pdf]

Table S1 Primer sequences used in this study.

Primer name	Primer sequence	
	Forward (5' to 3')	Reward (5' to 3')
shRNA (Oip5-as1)	GGAAAGACGGTGTTTGCAAGT	ACTTGCAAACACCGTCTTTCC
	GGACAATGCTCACCCTGAACT	AGTTCAGGGTGAGCATTGTCC
	GGTGGACCCTGATGTGTAACT	AGTTACACATCAGGGTCCACC
siRNA (AKAP1)-1	CGGGAACAGUAUGGAUUCATT	UGAAUCCAUACUGUUCCCGTT
siRNA (AKAP1)-2	GAGGUGAUGACAACUUUGUTT	ACAAAGUUGUCAUCACCUCTT
siRNA (AKAP1)-3	CGUGGACUAUGGUGGAUAUTT	AUAUCCACCAUAGUCCACGTT
siRNA (negative control)	UUCUCCGAACGUGUCACGUTT	ACGUGACACGUUCGGAGAATT
Probe (Oip5-as1)	CATCTTCGCTCCTTCCCCTGTC	
	TAACAGGATGGAAGAACCAGGACGC	
	CCAATTCAAAAGCTGCAGAAGACCC	
Probe (negative control)	TGCTTTGCACGGTAACGCCTGTTTT	
Probe (18S)	CTGCCTTCCTTGGATGTGGTAGCCGTTTC	
Oip5-as1	AGACTCAACACAGGAAAGCCG	CAACTCACGCCACAGAACCTA
Actb	GCTGCGTTTTACACCCTTTCT	TGCTCCAACCAACTGCTGTC
Neat1	TGGCCCCTTTTGTTCATTAGC	TGGAAGGCCATTGTTTCAGG
AKAP1	CAAAGGTAGCAGCAATACTTCG	CTTCAGTTGCTTCCAAGATCAC
Oip5-as1 floxed allele	GATTTCTGTCTGTTCTCCCTACCC	TATGACAGATCATAACACAGGGCA
Cre recombinase	GAAATGACAGACAGATCCCTCCTATC	CGACGATGAAGCATGTTTAGCTG

Table S2 Information of antibodies used in this study.

Antibody	Company	Catalog Number
AKAP1 (D9C5) Rabbit mAb	Cell Signaling Technology	5203
Anti-Bax Antibody	Abcam	ab32503
Anti-COX IV Mouse Monoclonal Antibody	Abbkine Scientific	A01060
Bcl2 Polyclonal antibody	Proteintech Group	26593-1-AP
Cleaved Caspase-3 (Asp175) Antibody	Cell Signaling Technology	9661
Cytochrome c (D18C7) Rabbit mAb	Cell Signaling Technology	11940
DRP1 (D6C7) Rabbit mAb	Cell Signaling Technology	8570
Pan-Calcineurin A Antibody	Cell Signaling Technology	2614
Phospho-DRP1 (Ser616) (D9A1) Rabbit mAb	Cell Signaling Technology	63940
Phospho-DRP1 (Ser637) Antibody	Cell Signaling Technology	4867
α/β-Tubulin Antibody	Cell Signaling Technology	2148
