# Supplementary figures and images for: lncRNA Oip5-as1 inhibits excessive mitochondrial fission in myocardial ischemia/reperfusion injury by modulating DRP1 phosphorylation
Source: Cell Mol Biol Lett. 2024 May 14;29:72. doi: 10.1186/s11658-024-00588-4 (PMC11092055; doi:10.1186/s11658-024-00588-4)

**A**

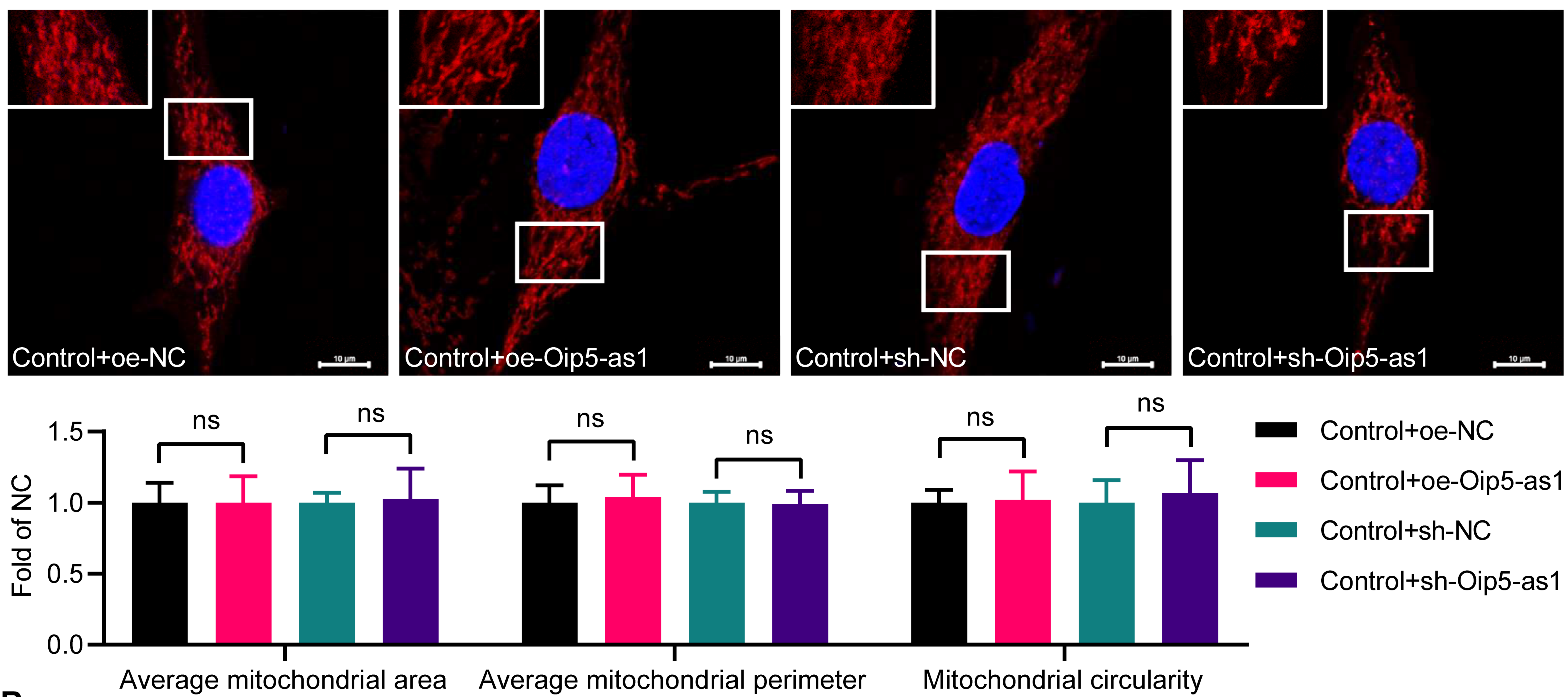

**B**

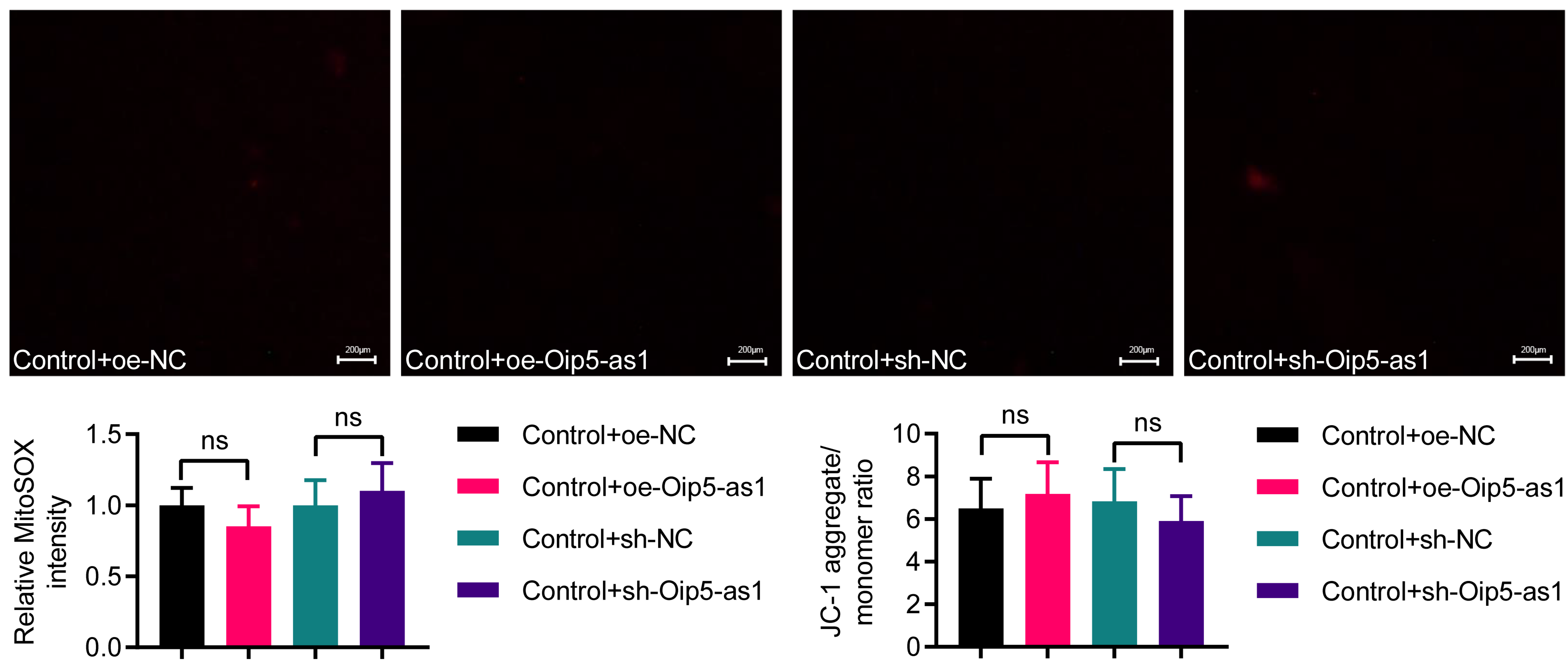

**C**

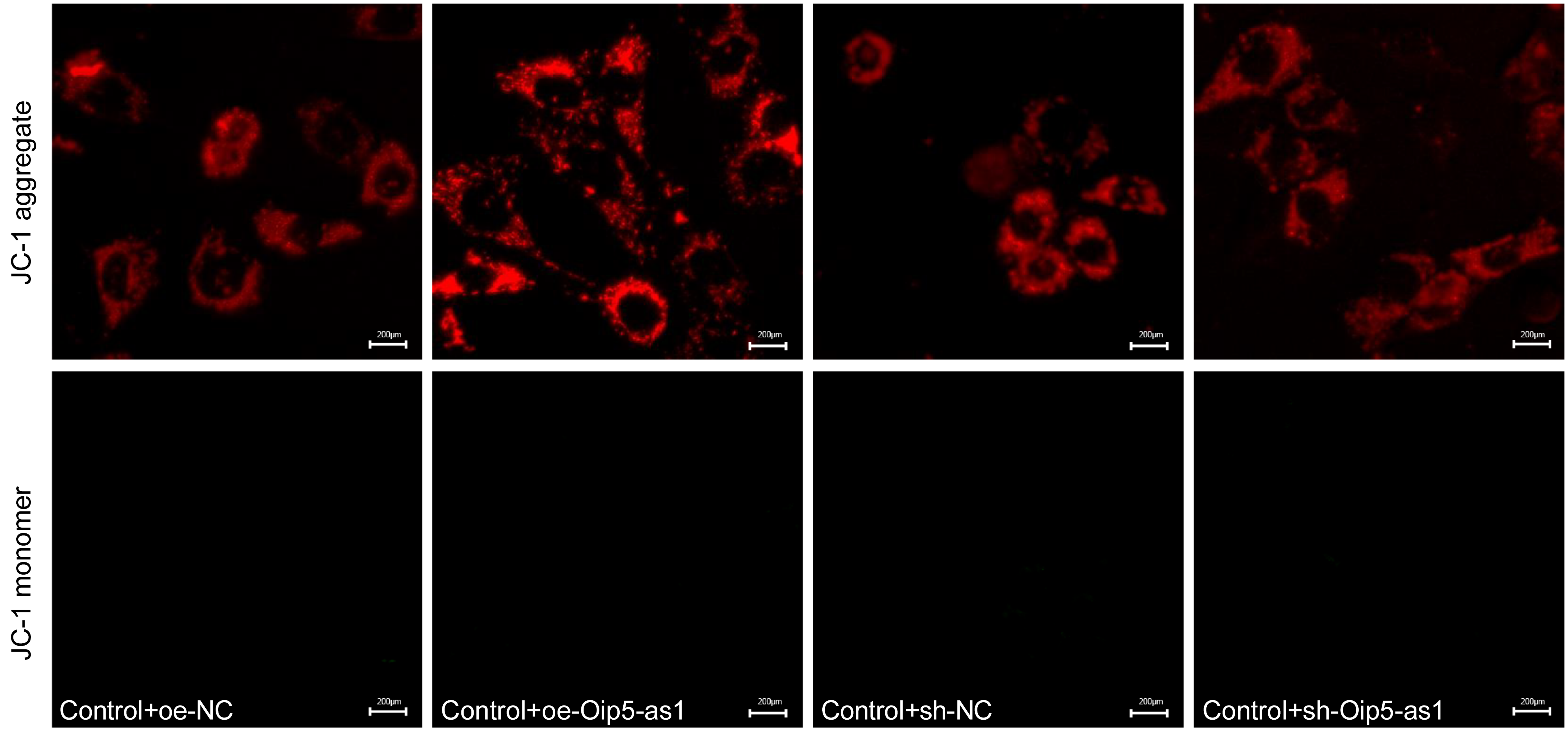

Supplement: Supplementary file 2 — Additional file 2: Fig. S1 Effects of Oip5-as1 on mitochondrial dynamics in HL-1 cells under normal culture conditions. A Mitochondrial morphology analysis of HL-1 cells transfected with oe-Oip5-as1 or sh-Oip5-as1. Data from representative images and quantification of Tom-20-stained HL-1 cells show no significant changes in mitochondrial morphology (average mitochondrial area, perimeter, and circularity) upon oe-Oip5-as1 or sh-Oip5-as1 transfection compared to oe-NC or sh-NC controls. The cell nuclei are stained with DAPI. Scale bar, 10 μm. B Mitochondrial-derived ROS levels in HL-1 cells transfected with oe-Oip5-as1 or sh-Oip5-as1. MitoSOX staining shows no significant differences in fluorescence intensity between oe-Oip5-as1 or sh-Oip5-as1 transfected groups and oe-NC or sh-NC controls. Scale bar, 200 μm. C Mitochondrial membrane potential changes in HL-1 cells transfected with oe-Oip5-as1 or sh-Oip5-as1. JC-1 staining shows no significant alterations in the aggregate/monomer fluorescence intensity ratio of HL-1 cells with oe-Oip5-as1 or sh-Oip5-as1 transfection compared to oe-NC or sh-NC controls. Scale bar, 200 μm. Data are presented as mean ± standard deviation, n = 3. ns nonstatistically significant. Tom-20 translocase of outer mitochondria 20. DAPI 4', 6-diamidino-2-phenylindole. ROS reactive oxygen species. oe-Oip5-as1 the overexpressed lentivirus for Oip5-as1. oe-NC the overexpressed lentivirus for negative control. sh-Oip5-as1 the short hairpin RNA (shRNA)-mediated lentivirus targeting Oip5-as1. sh-NC the negative control shRNA lentivirus [file 11658_2024_588_MOESM2_ESM.pdf]
